# Strategy of Triple‐Gradient in Binary Pixels for Flexible Pressure Sensing with High Sensitivity and Wide‐Range Linearity

**DOI:** 10.1002/advs.76572

**Published:** 2026-07-14

**Authors:** Yifan Liu, Xiao Xu, Fengming Hu, Yuanzhe Liang, Zidong Yin, Biao Qi, Ruolin Liu, Ziyi Dai, Jianyi Luo, Bingpu Zhou

**Affiliations:** ^1^ Joint Key Laboratory of the Ministry of Education, Institute of Applied Physics and Materials Engineering University of Macau, Avenida da Universidade Taipa Macau China; ^2^ School of Biomedical Engineering and Informatics Nanjing Medical University Nanjing China; ^3^ Research Center of Flexible Sensing Materials and Devices School of Applied Physics and Materials Wuyi University Jiangmen China; ^4^ Shanghai Key Lab. of D&A for Metal‐Functional Materials School of Materials Science & Engineering Tongji University Shanghai China; ^5^ School of Integrated Circuits Shandong University Jinan China

**Keywords:** binary pixel, gradient design, human‐machine interactions, machine learning, wearable sensors

## Abstract

Flexible pressure sensors have recently aroused increasing interests for fields of electronic skin, intelligent robotics, and human‐machine interactions (HMI). Preserving high sensitivity of flexible pressure sensors across a broad range is essential prerequisite to ensure signal‐to‐noise reliability for multiple applications. However, this remains challenging due to the inherent sensing saturation when flexible matrix is exposed to varying pressure. Herein, a flexible pressure sensor based on design of binary micro‐dome pixels is proposed. Triple‐gradient of conductivity, modulus, and dimension is precisely designed in binary pixels, ensuring mechanical and electrical compensation during the entire deformation process. With well‐developed CNT/PDMS matrix, significant sensing enhancement is realized, showcasing a linear sensitivity of 974.1 kPa^−1^ across range up to 1.8 MPa (R^2^ > 0.99). Thanks to the preserved sensitivity, the wearable device can recognize body information such as pulse, joint motion, and even foot pressure. The broad linearity also allows HMI establishment that applies force level as one basic clue to reflect human intension, e.g. dual‐factor authentication system. Along with superiorities of low detection limit, fast response, and mechanical robustness, the principle of triple‐gradient in binary pixels can be of significance for development of high‐performance flexible sensors in the future is expected.

## Graphical abstract

A flexible pressure sensor with triple‐gradient design of conductivity, modulus, and dimension in binary micro‐dome pixels is proposed. Based on precisely‐designed CNT/PDMS matrix, the device exhibits a linear sensitivity of 974.1 kPa^−1^ across range up to 1.8 MPa (R^2^ > 0.99), offering an effective strategy for potential applications in healthcare monitoring and wearable human‐machine interactions.

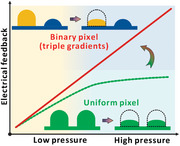

## 1 Introduction

Human skin, the largest organ of the body, accounts for approximately 15% of an adult's body weight and covers a total area of about 1.5 to 2 square meters [1, 2]. It not only perceives external stimuli and protects against harm but also serves as the primary interface for communication with the surrounding environment. Within human skin, a variety of nerves and receptors are distributed across different layers, each fulfilling specific roles. For instance, Meissner's corpuscles act as rapidly adapting receptors, capable of detecting light touch and enabling precise stimulus recognition and localization [3, 4]; meanwhile, Pacinian corpuscles can detect rapidly changing pressure and high‐frequency vibrations, allowing for the detection of deeper pressure variations [5, 6]. In recent years, flexible electronic skin (e‐skin) has emerged, aiming to simulate these intricate sensory mechanisms of human skin, striving to replicate or even surpass its perceptual functions. This is achieved by utilizing various sensors that convert physical stimuli into electrical signals, thereby enabling a biomimetic reproduction of skin functionality [7, 8, 9]. Owing to their ability to perceive and respond to a wide range of stimuli, electronic skins have found extensive applications in wearable health monitoring [10, 11], rehabilitation therapy [12, 13], human‐machine interaction [14, 15], artificial intelligence [16, 17], and soft robotics [18, 19]. Among the various sensory functions, touch is the most fundamental and critically important aspect. Correspondingly, pressure sensors play a key role in enabling tactile perception within electronic skin systems. Therefore, the development of high‐performance pressure sensors is crucial for the overall sensory system, rendering potential to mimic the sensing capability toward different applications.

Pressure sensors can generally be categorized into several types based on their signal conversion mechanisms, including resistive [20, 21], capacitive [22], piezoelectric [23], and triboelectric [24] sensors. Among these, flexible resistive pressure sensors have garnered significant attention due to their advantages of low power consumption, broad material compatibility, good mechanical adaptability, simple fabrication processes, and low cost [25, 26, 27]. However, the trade‐off between high sensitivity and a wide linear range remains a core challenge in this field, and achieving a synergistic optimization of both is a critical issue that needs to be addressed. To address this challenge, researchers typically focus on material innovation and structural design to enhance the overall performance of flexible pressure sensors. In terms of materials, common strategies include the incorporation of novel functional materials such as two‐dimensional transition metal carbides (MXene) [28, 29], conductive polymers (e.g., PEDOT:PSS) [30, 31], and liquid metals (e.g., gallium‐indium alloys) [32, 33]. Regarding structural design, techniques such as surface microstructure modification (e.g., micropyramids [34, 35], microdomes [36], and microcolumns [37, 38]) or porous structure [16, 39, 40] design are widely employed. However, traditional single microstructure often provides high sensitivity in low‐pressure ranges, but quickly exhibits saturation with increased pressure that finally results in a limited linear range.

In view of this, we herein developed a flexible resistive sensor based on conductive carbon nanotubes/polydimethylsiloxane (CNT/PDMS) by controlling triple‐gradient effects of the conductivity (σ), elastic modulus (E), and geometry/height (H) within a binary unit to optimize the sensitivity and linearity range. Via precisely regulating the triple‐gradient within binary micro‐dome structures, the tactile device reveals a simultaneous enhancement in both sensitivity (974.1 kPa^−^^1^) and linear range (1.8 MPa). Furthermore, the sensor exhibits a low detection limit of ≈1 Pa, fast response of 15/16 ms, and maintains excellent mechanical and electrical stability in fatigue tests up to around 20 000 cycles. Due to the preservation of ultra‐high sensitivity across a broad pressure range, it can capture a wide array of human physiological signals from pulse beats, throat swallowing, and larger physiological signals generated during high‐intensity activities such as walking and running. Additionally, we successfully implemented the interface of Morse code, significantly improving the information transmission efficiency compared to traditional time‐defined methodology. By integrating machine learning, handwritten digital recognition with an extremely high accuracy of 99% was achieved. Given the sensor's extensive sensing range, we specifically designed an insole system to real‐time monitor foot pressure distribution and demonstrated the possibility of dual‐factor authentication system for password determination. Along with the proof‐of‐concept demonstrations, we expect the principle of triple‐gradient in binary pixels can provide instructive information to develop high‐performance flexible pressure sensors in the future.

## 2 Results and Discussion

### 2.1 Mechanism and Optimization Principle

**FIGURE 1:**
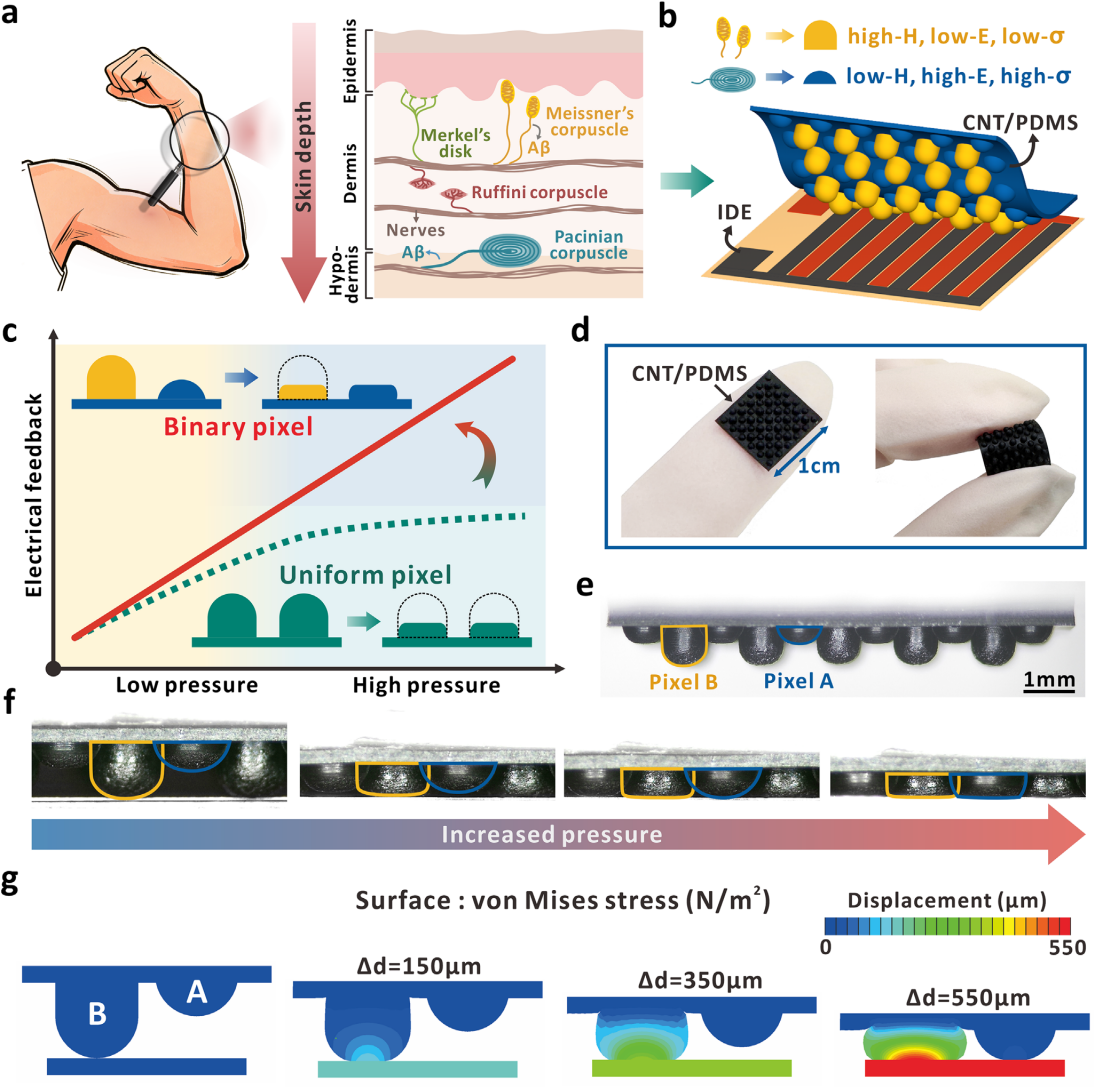
(a) Distribution of human sensory receptors along skin depth. (b) Illustration of the bio‐inspired triple‐gradient based flexible sensor. (c) Comparison of sensing process between triple‐gradient based binary pixel and non‐gradient based uniform pixel. (d) Optical image of the resistive layer in general views. (e) Optical image of the resistive layer in cross‐sectional view. (f) Optical images of the gradient micro‐dome structures when exposed to continuously increasing pressure. (g) Simulation results of force distributions in the binary pixels during compression. The heights of the micro‐domes in the binary pixel are 900 µm (B) and 400 µm (A), respectively.

Human skin is composed of three layers: epidermis, dermis and hypodermis. Each layer contains distinct sensory nerve endings and receptors. Specifically, Meissner's corpuscles can detect subtle stimuli and low‐frequency vibrations, such as the sensation of silk gliding over the skin, whereas Pacinian corpuscles respond to deep pressure and high‐frequency vibrations, such as intense impacts. As illustrated in Figure 1a, Meissner's corpuscles are predominantly distributed in the superficial dermis (papillary layer), while Pacinian corpuscles are in the deep dermis (reticular layer) and parts of subcutaneous tissue. Enabled by such synergistic effect, human skin can perceive a broad spectrum of external mechanical stimuli with sensitive feedback. Inspired by the layered distribution of receptors and corpuscles, flexible pressure sensors using triple‐gradient sensing elements were designed herein to mimic the tactile perception. Through precise modulation of structural geometry (e.g. height, H), modulus (E), and conductivity (σ), collaborative optimization of linearity and sensitivity was enabled based on specialized design within a binary sensing pixel. Figure 1b provides the schematic design of the device, which consists of the flexible sensing layer that is directly applied to the interdigitated electrode (IDE) layer underneath. In the binary pixel design, Pixel B, characterized by a taller geometry (high‐H), lower elastic modulus (low‐E), and relatively lower conductivity (low‐σ), mimics the Meissner's corpuscles in the superficial skin layers. In contrast, Pixel A simulates the Pacinian corpuscles in deeper skin layers, featuring a shorter geometry (low‐H), higher elastic modulus (high‐E), and relatively better conductivity (high‐σ). With the well‐developed micro‐dome structures, the alignment of micro‐structure to the gaps between interdigitated electrodes allows the conductive area to be significantly varied when different pressure is applied.

Figure 1c further compares the sensing capability between uniform pixel design and binary pixels with triple‐gradient. For uniform pixel, the initial contact between all micro‐domes and the electrode results in significant contact areas and thus the electrical current. This accordingly decays the sensitivity, followed by sensing saturation due to the mechanical constraint and limited electrical compensation if larger pressure is applied. Instead, triple‐gradient design allows precise control of initial contact regions that can only exist between electrode and the higher micro‐dome pixel. With continuously increased pressure, the compression of higher micro‐dome finally results in the contact of smaller pixels. This effectively compensates for the saturated contact area and electrical pathways. Furthermore, the electrical and mechanical properties of individual pixel can be modified to regulate the signal variations. Consequently, the binary pixel design can maintain a linear sensing behavior across wide‐pressure range if the gradient parameters are optimized. Figure 1d,e present optical images of the sensing matrix from different angles, visually demonstrating the structural design and flexibility of the sensing element. The detailed fabrication process was provided in Experimental section and Figure S1. The micro‐domes are classified into two heights, both with a structural radius of 500 µm. The shorter structure is designated as A, while the taller structure is designated as B. Structure A consists of a 6 × 6 array, and structure B consists of a 5 × 5 array, arranged in an area of 1 × 1 cm^2^ with a thickness of approximately 150 µm on a planar conductive layer, where the material of the conductive layer is consistent with that of micro‐dome A. Figure S2 further provides the top‐view images that directly reflect the array of binary pixels, where pixels A and B were closely staggered within the matrix. The real‐time deformation of binary pixels was provided in Figure 1f, where the normal pressure was continuously increased to compress the micro‐domes. It can be observed that in the initial status, only Pixel B is in contact with the bottom substrate. When pressure is applied, Pixel B is compressed first according to the magnitude of the normal pressure. Once the displacement reaches a specific value (e.g. 500 µm), Pixel A begins to contact the bottom substrate. As pressure continues to increase, Pixel A also starts to compress and more contact areas are produced during the continuous deformation. The experimental results are consistent with the simulation profiles (Figure 1g). During the compression process, the force is primarily concentrated at the top of the micro‐dome structure and subsequently dissipates radially outward through the matrix. With continuously increased pressure, the significant deformation of Pixel B finally leads to the contact between Pixel A and the substrate. From this perspective, the mechanical gradient allows the regulation of contact area and deformation capability of the overall system, rending the possibility of optimizing sensing feedback that is associated with the applied pressure.

**FIGURE 2:**
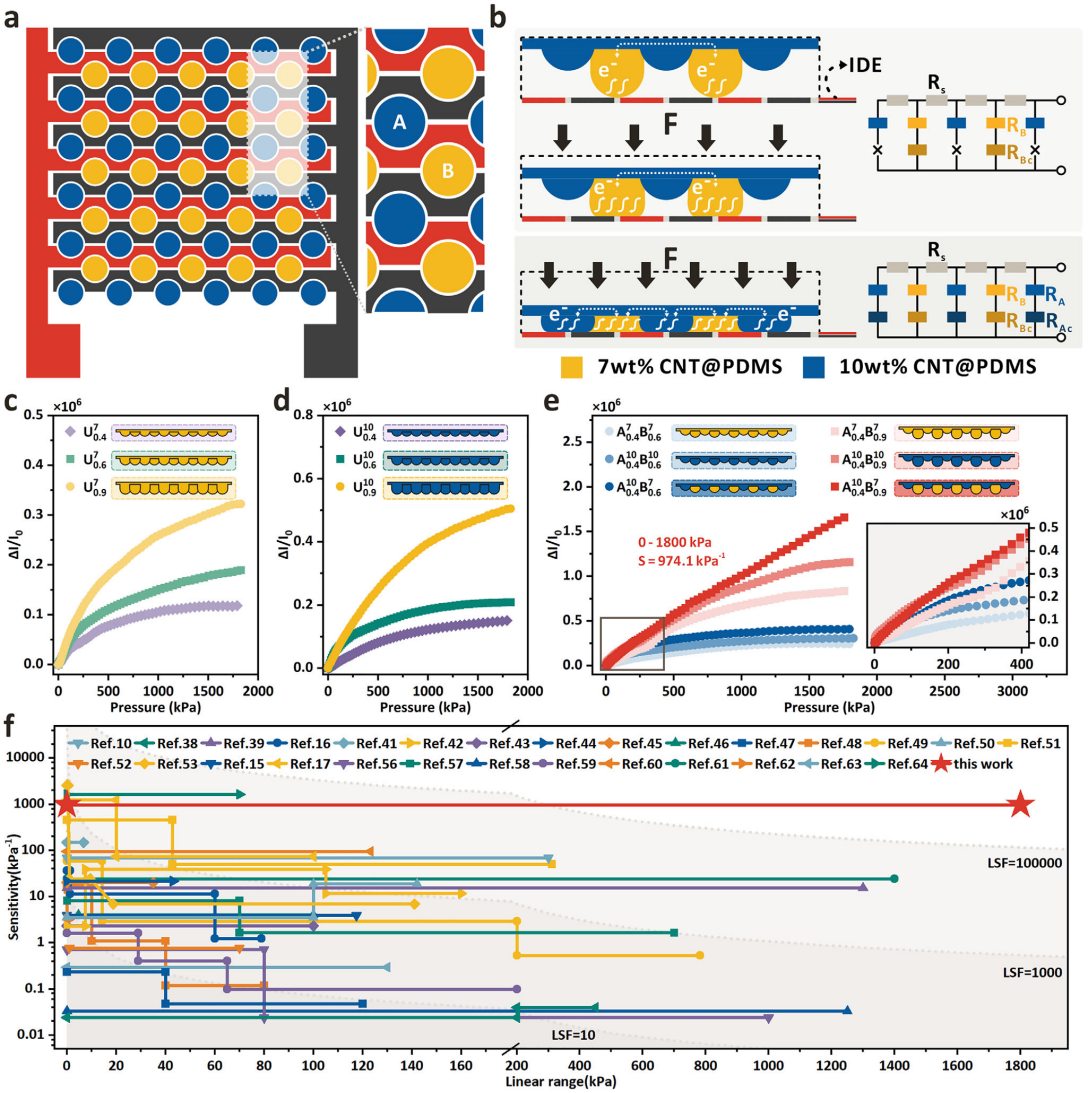
(a) The overall design of the proposed resistive pressure sensor. (b) Working mechanism of the resistive sensor by triple‐gradient effects from the conductivity, elastic modulus, and geometry. (c) The relative current change of devices with fixed conductive material mass fractions of 7 wt.%, U7 0.4, U7 0.6, U7 0.9 as a function of applied pressure. (d) The relative current change of devices with fixed conductive material mass fractions of 10 wt.%, U10 0.4, U10 0.6, U10 0.9 as a function of pressure variation. (e) Relative current change in the gradient‐based sensors A7 0.4B7 0.6, A7 0.4B7 0.9, A10 0.4B10 0.6, A10 0.4B10 0.9, A10 0.4B7 0.6, and A10 0.4B7 0.9. (f) Comparison results regarding the sensitivity and linear range among our proposed sensor and other reported flexible resistive sensors.

Figure 2a illustrates the detailed design of sensing architecture and the electrode layout, where the pixels A and B are arranged in staggered behavior for optimization of the sensor performance. As mentioned above, a triple‐gradient methodology within binary micro‐domes was introduced here to address the rapid sensing saturation of an individual micro‐dome when exposed to increasing pressure. We selected interdigitated electrodes for sensor evaluation due to the advantages including fast response time, simple structure, and ease of integration. Additionally, the geometric parameters of the interdigitated electrodes, such as finger spacing and finger width, are easily adjustable, rendering significant potential in optimizing the sensor's performance. The finger width of the interdigitated electrodes is 800 µm, with a finger spacing of 100 µm that is associated with the layout of the micro‐dome arrays (Figure S3). To maximize the electrode engagement under applied pressure, the apexes of the dome structures are strategically positioned between two interdigitated fingers. As shown in Figure S4, the initial current comparison of A10 0.4B7 0.9 and U7 0.9 reveals that U7 0.9 provides more domes contact to the circuit. This results in a greater number of parallel conducting pathways and a smaller resistance to induce larger initial current. Here, the superscript denotes the composition of conductive material and the subscript represents the height of the micro‐dome. For example, U7 0.9 means the micro‐domes were prepared with uniform height of 900 µm in a 7% mass percentage of CNT/PDMS. A10 0.4B7 0.9indicates the micro‐domes were arranged in binary mode, where Pixel A is with height of 400 µm, 10% ratio of CNT/PDMS and Pixel B is with height of 900 µm, 7% ratio of CNT/PDMS. In contrast, only domes with a height of 900 µm in A10 0.4B7 0.9 contact the electrode region, forming fewer parallel pathways and a higher resistance, thus the current is obviously smaller. Meanwhile, we also tested the stability of the electrode by bending it at different angles (0°–180°). After bending the electrodes up to 180°, the electrode resistance was able to recover to its initial value, demonstrating electrical stability after a complete bending cycle (Figure S5).

The detailed sensing principle of the sensor was provided in Figure 2b. Here, R_S_ represents the resistance of the bottom surface, R_A_ is the resistance of microstructure A, R_B_ is the resistance of microstructure B, R_AC_ is the contact resistance of microstructure A, and R_BC_ is the contact resistance of microstructure B. At lower pressures, only the taller microstructure B is connected to the circuit, and the adjacent microstructures B are connected in parallel to form several conductive pathways. As pressure increases, the deformation of structure B becomes more pronounced, leading to a decrease in R_BC_ and a corresponding increase in electrical current. Ultimately, with further increased pressure, the smaller microstructure A also initiates to engage with the circuit, and the adjacent microstructures A connect in parallel with the already engaged microstructures B. With continuously increased pressure, both R_AC_ and R_BC_ decrease, thereby reducing the overall circuit resistance and increasing the total current. Based on the arrangement, microstructure A consists of a CNT/PDMS mixture with stronger conductivity and higher elastic modulus, while microstructure B is made of a CNT/PDMS mixture with lower conductivity and elastic modulus. As demonstrated below, the triple‐gradient from geometrical heights, electrical conductivity, and elastic modulus contribute synergistically to preserved sensitivity across a broad pressure spectrum.

First, we investigated the influence of dome height on the performance of sensors fabricated using the same conductive material. As shown in Figure 2c, we designated devices with dome heights of 400 µm and a CNT/PDMS weight fraction of 7 wt% as U7 0.4. It can be observed that with a larger height of the micro‐domes, the sensitivity improves progressively and eventually reaches a saturation state. Here, the sensitivity (S) of the flexible sensor is defined as

$$S=\left(\frac{\Delta I}{I_{0}}\right)/\Delta P$$

where *I_0_* is the initial current without external load, and *ΔI* denotes the change in current under the applied pressure variation of *ΔP*. The sensitivity enhancement is attributed to the increased height of the sensing element, which provides a more significant space that allows the structural compression and decay of the overall resistance. However, all cases exhibit a non‐linear sensing performance, indicating the optimization through uniform geometry cannot be simply realized due to the synchronous compression of micro‐structures. Further, we explored the effect of the conductivity and modulus of micro‐domes when the geometry within the sensor is uniform (Figure 2d). With a higher weight percentage of CNT/PDMS, the conductivity and modulus can be simultaneously enhanced (Figure S6). When pressure increases, the compression of sensing micro‐domes results in a higher electrical current to reflect the applied stimuli. However, the sensing curve also exhibits a non‐linear trend when the applied pressure is continuously increased. The results reveal the challenge to maintain the sensitivity across a broad pressure range if based on single variable regulation.

Instead of uniform micro‐dome design, Figure 2e compares the sensing performance when multiple gradients were applied to the micro‐domes in binary architecture. When the configuration of micro‐dome heights was defined at 400 µm (A_0.4_) and 600 µm (B_0.6_), we further regulated the conductivity and modulus of each pixel in the array. Using a highly conductive composite for A and a less conductive composite for B (A10 0.4B7 0.6), the sensitivity was moderately improved when compared with the devices from identical conductivity and modulus (A7 0.4B7 0.6 and A10 0.4B10 0.6). However, the limited height difference is insufficient to provide additional conductive pathway during the application of high‐pressure stimuli. Considering this limitation, a more significant heigh difference between pixels A (A_0.4_) and B (B_0.9_) was introduced. Thanks to the triple‐gradient effect within the binary pixel, the specific configuration of A10 0.4B7 0.9showcases a wide linear range up to 1.8 MPa with a high sensitivity of 974.1 kPa^−1^. To further clarify the sensing process, real‐time record of the micro‐dome compression was provided in Figure S7. Compared with uniform geometry, binary micro‐dome structures exhibit different behavior according to the increase of normal pressure. For A_0.4_ B_0.6_, the 400 µm‐high dome begins to make contact at approximately 80 kPa, while for devices with heights A_0.4_ B_0.9_, contact occurs around 300 kPa. Upon contact, additional pathways are connected in parallel to the circuit. Such behavior can effectively reduce the overall resistance and increase the current, avoiding sensing saturation as presented in the sensing curve. For devices with a single dome height of 1200 µm, structural tilting of the domes is observed when pressure exceeds 500 kPa. Consequently, during the entire compression process, the current exhibits a trend of increasing to a certain point and then subsequently decreasing, as illustrated in Figure S8. As shown in the enlarged view on the right side of Figure 2e, the configuration A10 0.4B7 0.6 exhibits a sensitivity greater than that of A7 0.4B7 0.9 within the pressure range of 0 to 300 kPa. This is attributed to the fact that the material for the base of the former is a 10 wt% CNT/PDMS mixture, which possesses better conductivity, resulting in higher sensitivity at lower pressure levels. After reaching 300 kPa, the sensitivity gradually approaches saturation as the pressure increases. To verify that the observed differences are due to the variation in substrate materials, we fabricated devices with a PDMS substrate (keeping all other ratios and experimental conditions identical) for comparison, as shown in Figure S9. The devices with a non‐conductive substrate exhibited a smaller current change rate and lower sensitivity compared to those with a substrate identical to Pixel A, thereby confirming the differences as mentioned above. To further verify the essential role of triple‐gradient design, we exchanged the conductivity of Pixel A and Pixel B to monitor the sensing performance. As shown in Figure S10, the current‐change rate in A7 0.4B10 0.9 is greater than that in A10 0.4B7 0.9 when the applied pressure is below 300 kPa, indicating higher sensitivity within the investigated pressure range. For A7 0.4B10 0.9, Pixel B is composed of a CNT/PDMS (10 wt%) with higher conductivity when compared to Pixel A. Therefore, the current‐change range is larger and the sensitivity is higher under the same applied pressure. However, as the applied pressure continues to increase until Pixel A begins to contact the electrode, the current increases slowly and a gradual saturation behavior is observed. After exchanging the conductivity of Pixel A and Pixel B, the sensing curve exhibits a non‐linear trend that is dependent on the pressure window, confirming the necessity of the triple‐gradient design where conductivity ratio also plays an important role. Figure 2f finally summarizes the sensitivity and linear range of relevant piezoresistive pressure sensors [10, 15, 16, 17, 38, 39, 40, 41, 42, 43, 44, 45, 46, 47, 48, 49, 50, 51, 52, 53, 54, 55, 56, 57, 58, 59, 60, 61]. The sensors designed in this study demonstrate exceptional sensitivity with extensive linear range that produces a linear sensitivity factor (LSF) of 1 753 380 (the product of sensitivity and linear range). Detailed values of sensitivity, linearity range, detection limit, and materials for the electrodes and conductive layer are provided in Table S1.

### 2.2 Characterization of Sensing Performance and Wearable Applications

**FIGURE 3:**
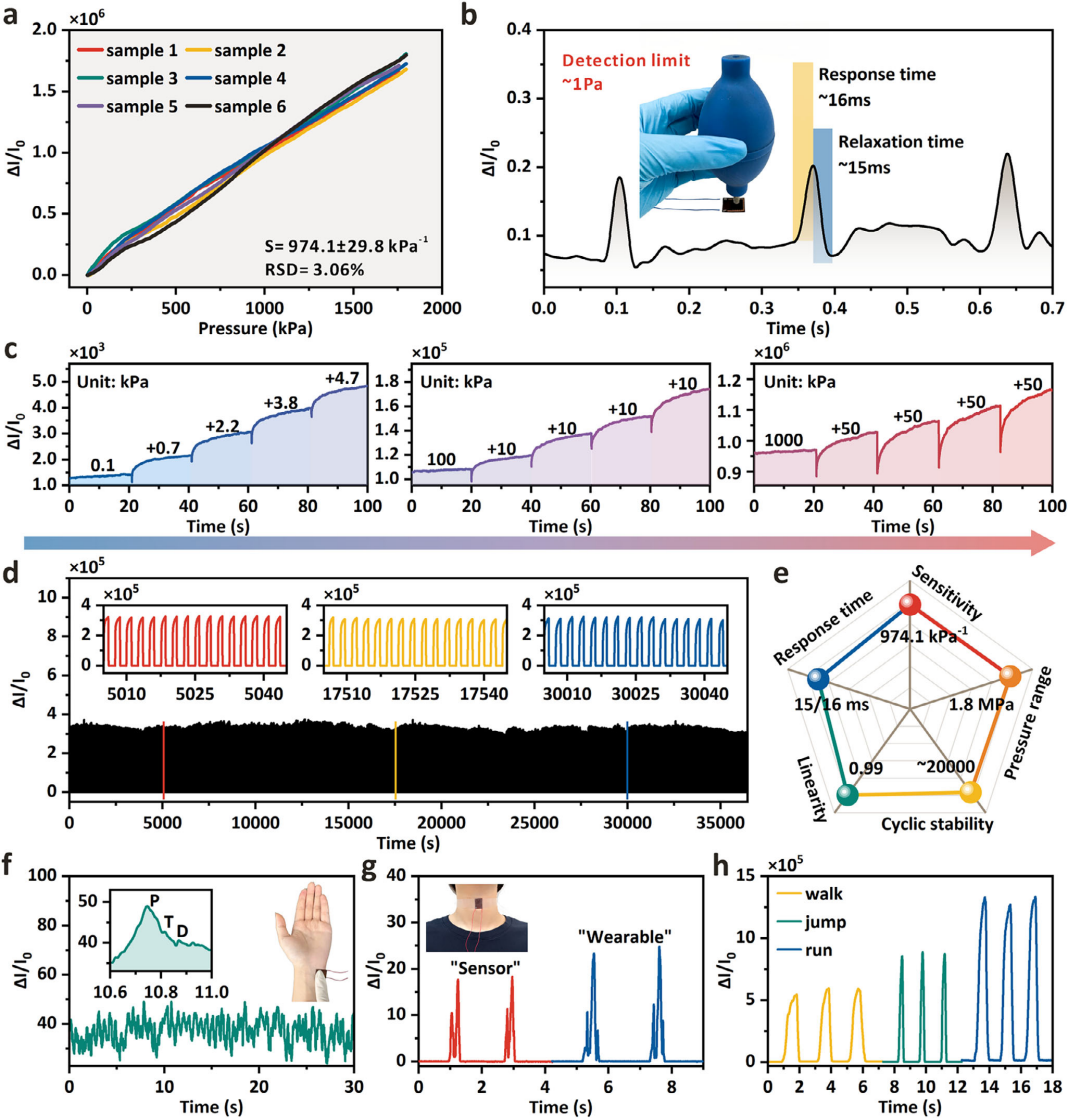
(a) Relative signal variations of the proposed sensor from different samples. (b) Fast response/relaxation time of the sensor and the experimental results of sensing detection limit. (c) Recognition of pressure variations of the proposed sensor when 0.1, 100, and 1000 kPa have been pre‐loaded. (d) Long‐term stability of the sensor under a periodic pressure of ≈350 kPa at a frequency of 0.5 Hz, under room temperature of ≈25°C and relative humidity of 67.4%. The insets show signals from 5000 to 5050 s, 17 500 to 17 550 s, and 30 000 to 30 050 s. (e) Radar chart of basic performance for the proposed sensor. (f) Real‐time response of the flexible sensor for wrist pulse measurement. (g) Real‐time monitoring of voice vibration in response to pronunciations of a bisyllable word “sensor” and a multisyllabic word “wearable”. (h) Dynamic response of the sensor attached on sole during daily walking, jumping and running.

We further investigated the sensing performance of the sensors from different aspects including sensing repeatability, responsive behavior, detection limit, and mechanical stability. Such parameters are essential to ensure reliable applications of flexible wearable sensors for home healthcare monitoring, human‐machine interactions, motion capture, and medical diagnosis. The repeatability of the sensor across different samples was evaluated in Figure 3a. For each dataset, the current values at specific pressure were obtained by averaging the signals when the applied pressure was maintained for 20 s. Within the pressure range of 0 to 1800 kPa, all samples exhibited excellent repeatability with an average sensitivity value of 974.1 kPa^−^^1^ (R^2^ > 0.99) and a standard deviation of 29.8 kPa^−^^1^. Consequently, the relative standard deviation (RSD) was calculated to be 3.06%, indicating the sensing reproducibility of the flexible sensor. We have provided error bars for the plot in Figure S11, which exhibits the consistency of electrical signals during the characterization process. Additionally, we also verified the performance and consistency of devices with parameters of A_0_._4_B_0_._7_ and A_0_._4_B_0_._8_ (Figures S12 and S13). Both configurations demonstrated favorable repeatability with R^2^ > 0.99. The loading and unloading curves of a typical device exhibit a high degree of overlap without significant hysteresis (Figure S14). This characteristic is crucial for maintaining the accuracy of physiological signal monitoring in wearable applications, ensuring the reliability of diagnosis and treatment upon mechanical loading and release.

The responding behavior of the flexible sensor was further measured based on dynamic pressure by manually squeezing a wash ball toward the device (Figure 3b). Upon receiving the pressure, the encapsulated device quickly provided electrical feedback to reflect the mechanical inputs. By analyzing the duration between baseline and the electrical peaks, the response time and relaxation time were determined as ≈16 and ≈15 ms, respectively. The results reveal that the flexible sensor can react swiftly according to the mechanical stimuli. Given that the typical duration of cyclic physiological signals, such as heartbeat and pulse, is generally in the order of seconds, this provides promising opportunity for detecting the healthcare signals. Using the pressure from wash ball, the minimum detectable pressure by the sensor was measured on the balance as 0.01 g. With the sensing window (1 × 1 cm^2^), the minimum detection limit was roughly ≈1 Pa, showcasing the detection capability of tiny pressure. Furthermore, by adjusting the downward speed of the displacement platform, we tested the device's response at different frequencies. As shown in Figure S15, the device can respond promptly within the frequency range of 0.25 to 1.07 Hz. Due to its wide linear range, this flexible sensor can accurately identify pressures of 0.1, 100, and 1000 kPa, as well as the gradual loading of lower pressures (Figure 3c). Even with a pre‐loading, the continuous pressure application can still be recognized as reflected from the current variation. To further validate the device's ultrawide detection range, we incrementally increased the pressure from 5 Pa to 1700 kPa. As illustrated in Figure S16, the device consistently exhibited excellent response curves. Next, we tested the mechanical stability of the device. We further performed continuous loading and unloading within a pressure range of 350 kPa for approximately 20 000 cycles (cycle of ≈2 s for up to ten hours). The inserts convince the robustness of the device for possibility of long‐term applications (Figure 3d). After fatigue test, no significant geometry variation was observed on the sensing structures. The flexible sensor could also provide identical electrical signals when exposed to the same periodical pressure (Figure S17). We further investigated the effects of environmental temperatures, as well as humidity, on the sensing performance of the sensor. First, we simulated responses at four temperature conditions, approximately 10°C, 40°C, 60 °C, and 80°C, using a cooling plate and a heating platform (Figure S18). Under the same periodical pressure, the sensor could maintain identical signals when the surrounding temperatures were changed. Furthermore, a customized system was built to simulate different humidity conditions from 20% to 99.9% (Figure S19). Throughout this process, the electrical current remained stable when a constant pressure was applied to the sensor. The basic performance of the developed sensor based on binary pixel with gradient design was further summarized in Figure 3e. Overall, the pressure sensor exhibits excellent sensing performance from sensitivity, linear range, to fundamental requirements such as response time, cyclic stability, etc.

Thanks to the ultra‐high sensitivity and ultrawide linear range, the sensor can detect a variety of human physiological signals, ranging from subtle pulse, breathing signals to low‐pressure speech, swallowing movements, and further to high‐pressure actions such as jumping and running. As shown in Figure 3f, we placed the encapsulated device on the radial artery of the wrist, applying gentle pressure with a fingertip. The device was able to stably capture real‐time pulse signals. The enlarged view in Figure 3f illustrates typical pulse peaks, such as percussion (P), tidal (T), and diastolic (D), indicating the significant potential of the developed sensing component for healthcare monitoring. In Figure S20a, we used a wash ball to periodically blow air onto the encapsulated sensor, which successfully detected the periodic airflow signals. In Figure 3g, the sensor was affixed to the throat of a participant that pronounces the bisyllabic word “sensor” and the multisyllabic word “wearable”. The device exhibited two characteristic peaks and three characteristic peaks, respectively, validating its ability to reflect the production of syllables. Additionally, as demonstrated in Figure S20b, the sensor can detect real‐time swallowing motion. For post‐stroke patients and the elderly, effectively monitoring the swallowing frequency and strength can significantly reduce the risk of choking and aspiration pneumonia, facilitating home health monitoring and risk mitigation [62]. Coupled with the patient's own condition, the wearable sensor can possibly enhance the analysis and assessment of their health status.

Beyond detecting subtle pressures, we also evaluated medium and high pressures. We fixed the sensor at the wrist joint to perform periodic wrist bending, where the device generated a consistent response due to the variation of contact area (Figure S20c). Furthermore, when the device was secured at the finger joint, the amplitude of the current changes increased with the degree of finger flexion. The contact areas between micro‐domes and the electrode layer were varied according to different bending degrees. Thus, the range of current changes can also reflect the magnitude of the applied bending on the device (Figure S20d). Given the sensor's wide linear range, it can accurately detect signals from relatively high pressures associated with walking, jumping, and running (Figure 3h). This capability is potentially beneficial for quantitatively assessing muscle strength and balance in post‐operative patients as well as applications in real‐time healthcare monitoring.

### 2.3 Human‐Machine Interfaces

**FIGURE 4:**
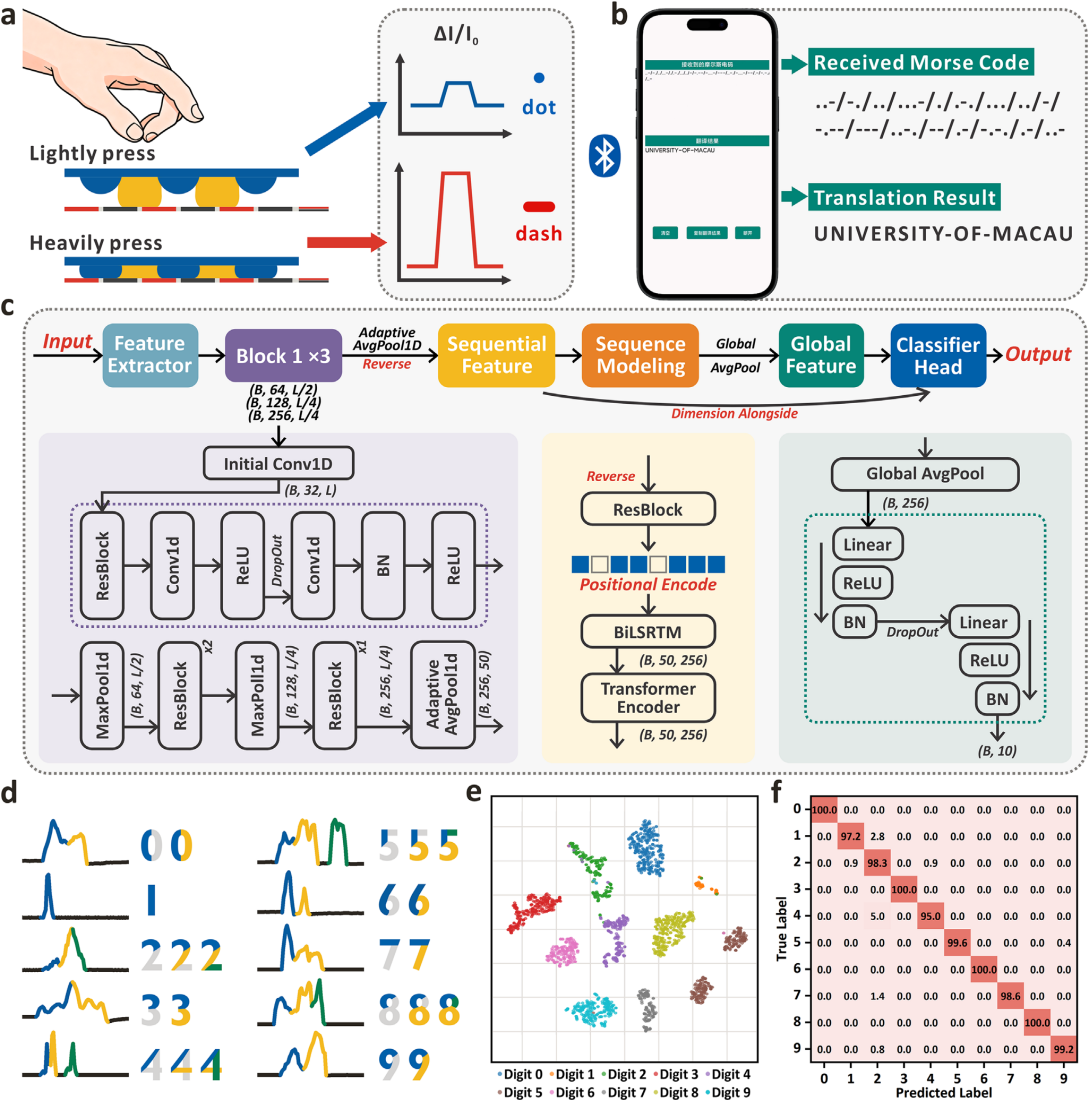
(a) Principle of Morse code generation based on the signal intensities by gentle tap or heavy press. (b) Demonstration of Morse code “UNIVERSITY‐OF‐MACAU” based on the flexible pressure sensor, where the electrical signals were transmitted by the customized circuit with Bluetooth wireless communication to the smartphone display. (c) Conceptual schematic diagram of machine learning network architecture based on HSS‐Net. (d) Detailed process and the real‐time electrical waveforms during writing numbers of 0 to 9. (e) t‐SNE visualization of high‐dimensional latent features from the test set containing digits 0 to 9. (f) The confusion matrix of our model for the 0 to 9 classification tasks.

Morse code can be utilized for information encoding through two fundamental signal units: dots and dashes. By employing various combinations and arrangements of these units, it can represent different letters, numbers, and symbols. Compared to traditional text, the primary advantage of Morse code lies in simplicity to enable global communication through basic dot and dash signals. In the domain of flexible pressure sensors, Morse code presents as a concise human‐machine interaction interface, showcasing the potential in wearable technologies and personalized interactions. Typically, dots and dashes were distinguished by the duration of the signals when flexible pressure sensor was exposed to mechanical stimuli [63, 64]. However, users may find this method challenging to accurately determine the start and end of pressure, leading to issues such as feedback delays and encoding errors during real operation. Given the wide linear range of our sensors, we can encode dots and dashes based on pressure intensity (Figure 4a), e.g. low pressure (< 150 kPa) is designated as a dot, while high pressure (>150 kPa, <1800 kPa) is designated as a dash. Based on this principle, we constructed a conversion methodology (Figure S21) for the customized circuit board with accompanying explanations. Figure S22 illustrates the system architecture of the Morse code circuit. The arrows indicate the direction of data transmission from the sensors to the electrical terminal. Within the same word, different letters are separated by “/” where the trigger condition for “/” is a pause with duration of ≈3 s. Between different words, separation is achieved using “—” with its trigger condition defined as the combination of one heavy press, four consecutive gentle taps, and another heavy press. As demonstrated in Figure 4b, the electrical terminal (mobile phone) could accurately receive the inputs and translate them into “UNIVERSITY—OF—MACAU” on the display via a Bluetooth module (see Videos S1 and S2 for complete communication procedures). Owing to the sensor's ultrawide linear range and exceptional stability, it enables reliable output of extended content. Compared to traditional Morse code defined by signal duration, our sensor allows the Morse code transmission in a more effective behavior within the same timeframe.

Benefiting from the triple‐gradient structural optimization, our sensor possesses distinct electrical and mechanical properties to maintain the sensitivity in each pressurizing stage, enabling the possibility for applications in handwritten digital recognition. As a proof‐of‐concept, we collected a dataset consisting of the digits from 0 to 9, with each digit represented by 1000 samples, resulting in a total of 10 000 data points. The dataset is split into training, validation, and test sets in a ratio of 7:2:1, ensuring reliable model selection and performance evaluation. Figure 4c illustrates the principles of machine learning. Within the overall system, the deep learning component is implemented as a one‐dimensional classification network specifically designed for handwritten digit recognition from current signals, referred to as the Hierarchical Stroke‐Sequence Network (HSS‐Net). As shown in Figure S23, as the training epochs increase, the trends of train loss and validation loss, train accuracy and validation accuracy align closely, indicating significant training effectiveness. The experimental results in Figure S23b indicate that the model achieved a high accuracy of over 99% on the test set.

Due to the unique stroke patterns of each digit and the varying contact points with the sensor, the resulting waveforms also differ. Figure 4d displays the writing process for digits 0 to 9, where the electrical signals provide typical waveforms according to the specific digits during writing. Figure 4e presents a t‐SNE plot for the ten digits processed through machine learning, demonstrating that the feature layer effectively distinguishes the ten digits, exhibiting excellent overall separability. A small number of sample points show actual misclassifications, which are consistent with the metrics from large‐scale data classification results. The confusion matrix illustrates the recognition accuracy for different digits throughout the entire testing process (Figure 4f). Compared to conventional writings, e.g. on paper, our flexible pressure sensor captures the two‐dimensional contours of handwriting while also recording pressure variations. In the information age, this technology could be developed into a dynamic biometric signature system that is difficult to replicate, suitable for high‐security signature authentication activities [65, 66]. Furthermore, when integrated with a password lock, it can achieve dual authentication for password correctness and the owner's biometric features, significantly enhancing security. In the medical field, patients with Parkinson's disease often exhibit tremors in their handwriting. Due to the ultra‐high sensitivity of our sensor, it can perfectly capture these subtle variations in writing, providing reliable data for early diagnosis and disease monitoring. Additionally, it holds significant value in assessing the recovery of patients’ hand motor skills by pressure tracking during real‐time writing.

**FIGURE 5:**
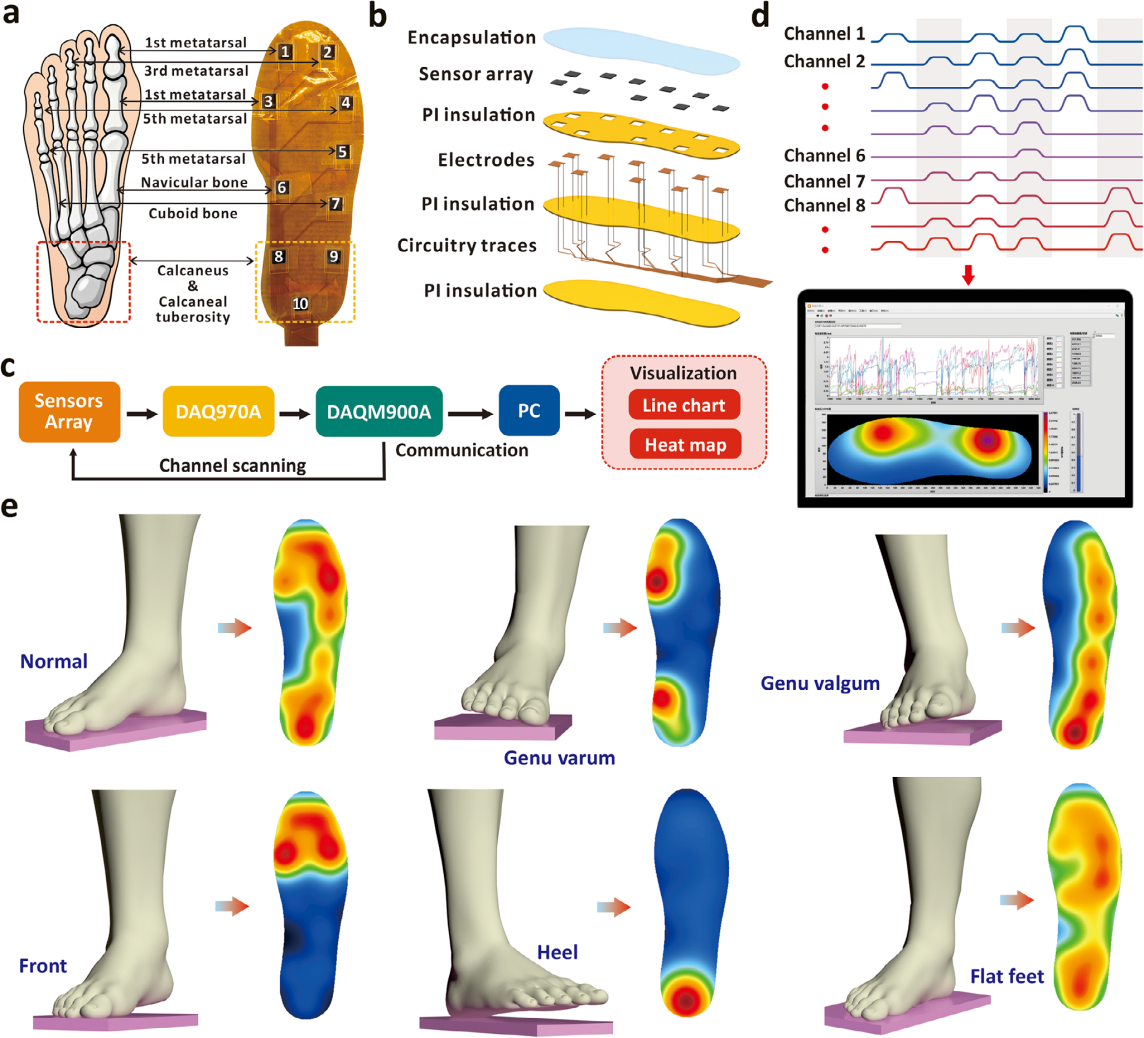
(a) Schematic diagram of resistive pressure sensor distribution on the insole. (b) Schematic diagram of the layered structure of the electrical insole. (c) System architecture of the insole system from signal generation to visualization. (d) Corresponding host computer display interface that relates to the signals from different sensor channels. (e) Schematic diagrams and plantar pressure distributions for normal stance, genu varum stance, genu valgum stance, toe, heel contact and flat feet.

Foot pressure and gait not only reflect abnormalities in the human locomotor system but also serve as important means for assessing the neurological system, metabolic system, and potential disease risks [67, 68]. Therefore, continuous monitoring of foot pressure and gait in daily life holds immense potential for the prevention, diagnosis, and treatment of various diseases, making it significantly important for health monitoring. An accurate foot pressure distribution map can visually indicate the presence of issues such as flat feet, high arches, and plantar fasciitis. Implementing real‐time monitoring of foot pressure and making timely adjustments to gait has a significant impact on addressing the issues such as excessive muscle fatigue, plantar fasciitis, and foot ulcers, etc. Herein, we designed an electrical insole to monitor real‐time plantar pressure. Based on the distribution of skeletal muscles and nerves in the foot, the insole is equipped with ten identical flexible pressure sensors (numbered 1 to 10). The sensors are distributed from the front to the back to convert the distribution of pressure signals into electrical signals. These sensors provide comprehensive coverage of the three main weight‐bearing areas of the foot: the forefoot, arch, and hindfoot. The forefoot and hindfoot typically exhibit more complex pressure patterns, leading to a denser arrangement of sensors in these regions to ensure the effective capture of key information with a limited number of sensors (Figure 5a). As shown in Figure 5b, the insole comprises upper and lower insulating packaging layers (adhesive stacking layer and PI film), conductive copper layers on both sides, and an internal polyimide (PI) layer. Figure 5c describes the design principle of the insole, by which the sensor signals can be transmitted to the terminal for real visualization. Based on signal collection from multiple channels, the pressure distribution on the insole can be determined in real‐time. The forefoot is corresponding primarily to the metatarsal region (sensors 1–5). During normal standing and walking, the primary weight‐bearing load is on the first and second metatarsals, which then transmit to the calcaneal region at the back of the foot. The arch is represented by sensors 6 and 7, strategically placed on the inner and outer sides of the arch. This arch structure aids in the distribution of pressure and can prevent overloading of individual metatarsals. Consequently, individuals with flat feet often experience greater fatigue, discomfort, and are more susceptible to conditions such as plantar fasciitis and hallux valgus. The hindfoot corresponds to the calcaneal region (sensors 8–10), which is the largest weight‐bearing bone in the foot to provide stable support. Each sensor operates as an independent channel, displaying the force experienced at the corresponding location when subjected to pressure. The electrical channels of 1–10 can record the pressure and convert to signals that finally generates the waveform or heat map for visualization of the foot pressure (Figure 5d).

Figure 5e presents heat maps under static conditions for a subject in six different postures: normal standing, genu varum (bow‐legged), genu valgum (knock‐kneed), tiptoe, heel strike, and flat feet. Different standing statuses of the volunteer were provided in Figure S24. In the first five postures, the insole is placed on the ground, simulating the direct pressure of the foot when standing. During normal standing, the plantar pressure is evenly distributed between the forefoot and hindfoot, with no significant pressure detected in the arch area. In the genu varum posture, commonly referred to as bow‐legged, the pressure is primarily concentrated on the inner side of the forefoot. Conversely, in the genu valgum posture, commonly known as knock‐kneed, the pressure is mainly distributed on the outer side of the forefoot. When in a tiptoe position, the center of gravity shifts forward, resulting in concentrated pressure on the forefoot. During heel strike, the center of gravity moves backward, with pressure predominantly on the hindfoot. Under normal circumstances, the arch section does not experience significant pressure. To simulate the condition of flat feet, we placed a thin board over the insole before standing on it (Figure S25), allowing uniform pressure distribution across all points on the insole. The resulting heat map indicates noticeable pressure in the arch area. In Videos S3 and S4, we demonstrated the insole fitted inside a shoe while simulating the aforementioned postures for real‐time monitoring. Note that due to the insole's fit within the shoe not being optimal, the simulation of certain postures is not as effective as when the insole is placed directly on the ground. When the insole is inserted into the shoe, there is closer contact between the foot and the insole, which causes some pressure to be exerted on the forefoot even during heel strike. Consequently, the heat map in the video shows some areas of the forefoot displaying color that gradually disappear. During normal standing with insole inside the shoe, the shoe may not conform tightly to the insole, leading to minimal pressure on the outer arch area. This results in lower current readings, and consequently no significant coloration is observed on the heat map. However, the real‐time record of foot pressure still reveals the potential of the flexible sensor for high pressure monitoring thanks to the wide linear working range.

In today's rapidly advancing information technology landscape, traditional digital passwords often pose security risks due to being susceptible to peeping or cracking. In view of this, we propose a dual‐authentication method that combines digital passwords with flexible pressure sensors. This approach integrates both the location mapping and applied pressure for enhanced security. Achieving this integration requires high‐performance devices with a broad linear range that respond promptly to stimuli, where the sensing resolution across different pressure range should be preserved. As a proof‐of‐concept, we constructed a 3×4 flexible pressure sensor array, representing the digits 0–9 as well as the symbols * and #. Figure S26 illustrates the working principle and signal processing of the dual‐factor authentication system. The dual‐factor decryption of the password can be successfully realized if the digits are correct and the corresponding pressures are accurately applied. Given the ultra‐high sensitivity and wide linear range of the device, we categorized the pressures into three levels: light, medium, and heavy. Therefore, the original 10^4^ combinations expand dramatically to (10*3)^4^ combinations for a four‐digit password, resulting in an 81‐fold increase in complexity and significantly enhancing the un‐locking difficulty.

**FIGURE 6:**
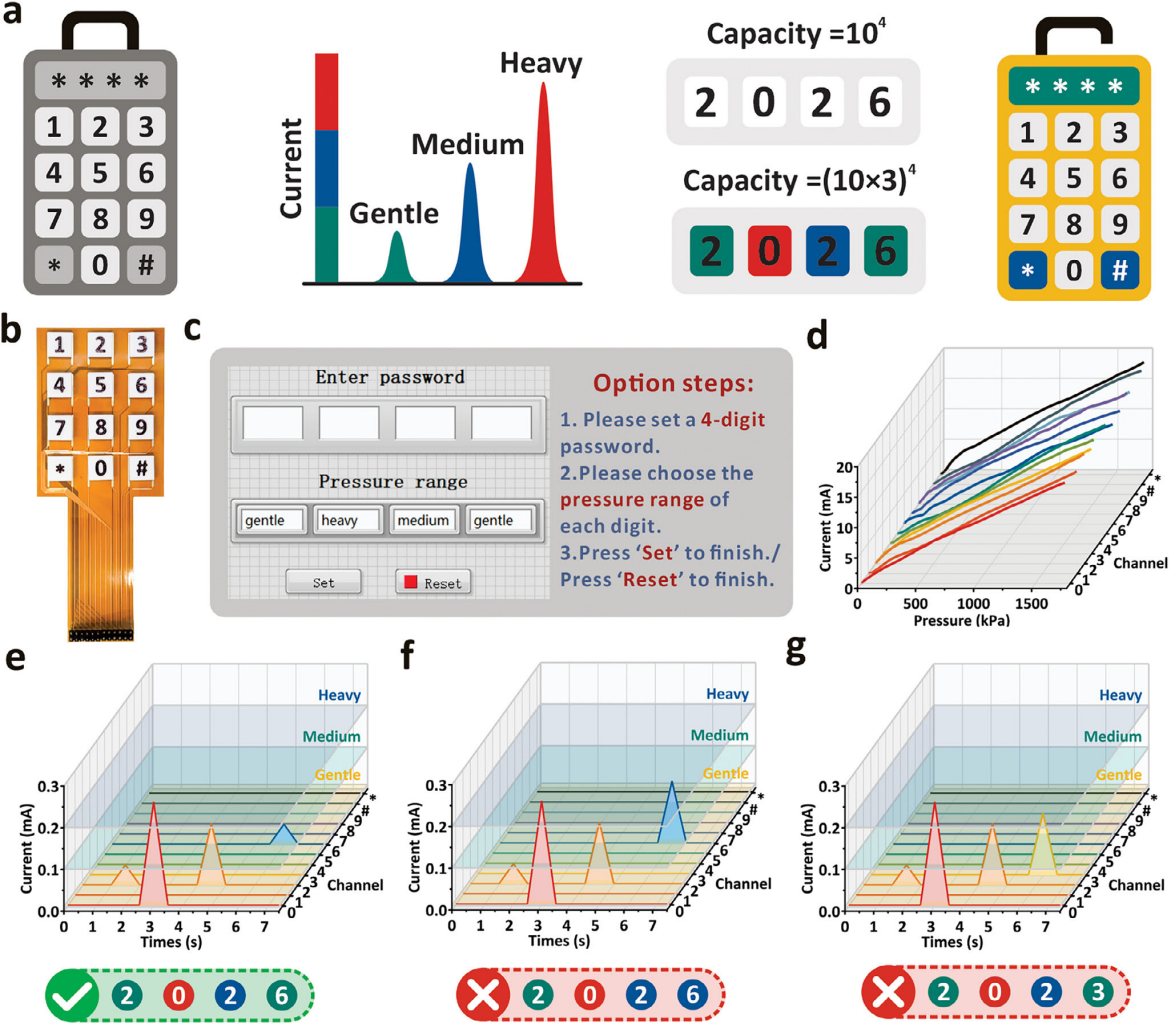
(a) Diagram of dual‐factor authentication passwords and definition of the typical signals from gentle press, medium press, to heavy press. (b) Electrode layout and input arrangement of the dual‐factor authentication system. (c) User interface of the dual‐factor authentication system. (d) Current variation of each channel of the sensor array in the authentication interface. (e) Correct current signal corresponding to the configured password. (f) Current signal of an input with correct numerical password but wrong press force information. (g) Current signal of an input with correct press force but wrong numerical password information.

Building on this concept, we simulated a reasonable arrangement of digits and symbols akin to a mobile phone dialing keypad. Figure 6b shows the physical representation of the dual‐authentication password system. Users can set a four‐digit password according to their preferences and select the corresponding pressure range for each digit (gentle, medium, heavy) from a dropdown menu. Figure 6c illustrates the specific operation interface for password setting. Each sensor constitutes a separate channel, and their responses to pressure signals are depicted in Figure 6d. Each channel maintains a high level of consistency in its response and exhibits good linearity across a wide response range, ensuring the accuracy and reliability of password reception and processing. We then demonstrated several scenarios: a correct password resulting in a successful unlock, and an incorrect password maintaining the lock state. Initially, we preset the password to 2026, where 2 corresponds to light press, 0 to heavy press, 2 to medium press, and 6 to light press. Light press is indicated by green, medium by blue, and heavy by red. The password‐setting process is recorded in Video S5. As shown in Figure 6e, we applied light press to 2, heavy press to 0, medium press to 2, and light press to 6, aligning perfectly with our preset digits and their corresponding pressures (see Video S6 for real‐time record). With matched pressure intensity and location (number) mapping, the password is correct and the system can be successfully unlocked. In contrast, as illustrated in Figure 6f and Video S7, we applied light press to 2, heavy press to 0, medium press to 2, and medium press to 6. While the digit passwords matched the preset values, the incorrect pressure intensity for digit 6 led to an incorrect password to remain a locked state. Similarly, we applied light press to 2, heavy press to 0, medium press to 2, and light press to 3 for password unlocking (Figure 6g and Video S8). Even though the pressures for each digit matched our preset values, the incorrect digit mapping failed to unlock the system. The results experimentally present the high security of authentication interface based on dual‐factor methodology.

## 3 Conclusions

In this work, via precisely defining triple‐gradient effect from the conductivity, elastic modulus, and geometry, we demonstrate the formation of flexible pressure sensors with a synergistic enhancement of linear range and sensitivity. Based on CNT/PDMS sensing element, the optimized sensor exhibits a high sensitivity of 974.1 kPa^−1^ over a wide linear pressure range of 0–1800 kPa (R^2^ > 0.99). Given the ultra‐high sensitivity and extremely wide detection range, the device can monitor physiological signals such as wrist pulse and voice vibration. Furthermore, it can potentially serve as a human‐machine interface for Morse code communication, real‐time gait monitoring, and a dual‐factor authentication system based on wide‐range pressure sensing. Thanks to the superiorities of fast response, low detection limit and mechanical robustness, the flexible device exhibits promising future for applications in areas such as postoperative monitoring, human‐machine interactions and soft robotics. We expect the principle of triple‐gradient optimization within binary sensing pixel opens avenues for design of high‐performance flexible pressure sensors in the future.

## 4 Experimental

### 4.1 Materials

The Polydimethylsiloxane (PDMS) base and curing agent (Sylgard 184 kit) were obtained from Dow Corning, USA, and are typically mixed thoroughly in a 10:1 mass ratio. The multi‐walled carbon nanotubes (CNTs), used as conductive materials, were purchased from Shenzhen Hongdachang Science and Technology Co. Ltd., China. Cyclohexane (analytical reagent), which serves as a solvent during the mixing process, was supplied by Tianjin Damao Chemical Reagent Factory, China. Additionally, the copper substrate, employed for interdigital electrode preparation, was purchased from Chengdu Do‐itc New Material Co., Ltd., China.

### 4.2 Fabrication Process of the Acrylic Template

The acrylic template used in this work was fabricated via micro‐engraving technology. Initially, a 100 µm‐deep groove was milled onto a 5 cm × 5 cm acrylic plate using a milling cutter. Subsequently, micro‐domes of varying heights (400, 600, and 900 µm) were carved within the grooves using a spherical cutter with a radius of 500 µm. In this study, the radius of the micro‐domes was fixed due to the geometrical limitation of the milling cutter. Alternatively, the heights of the micro‐domes were regulated to control geometrical gradient for sensing investigation. The different heights of the micro‐domes were achieved by adjusting the engraving depth of the milling machine accordingly. The concave structures were with bottom radius of 500 µm and edge distance of 800 µm.

### 4.3 Preparation of CNT/PDMS Conductive Layer

To prepare CNT/PDMS solutions with varying conductivities, precisely measured amounts (0.7 g for the 7 wt.% mixture, 1 g for the 10 wt.% mixture) of CNTs were dissolved in cyclohexane (30 mL) and stirred completely to make a homogeneous mixture. The solutions were ultrasonicated for 10 min and added to PDMS gel (9.3 g for the 7 wt.% mixture, 9 g for the 10 wt.% mixture), followed by a standard magnetic stirring for ≈12 h to completely evaporate the solvent under room temperature. Then, the mixture was added to standard PDMS curing agent in a typical mass ratio of 10:1. First, the mixture containing 7 wt.% CNTs was filled into the acrylic template and scraped repeatedly with a glass plate to ensure full filling with a flat substrate. Subsequently, the filled template underwent vacuum degassing to remove all air bubbles trapped within the patterned features. After complete degas process, the mixture from the groove surfaces was carefully wiped off using a tissue. Finally, the assembly was moved to oven (80°C) for approximately 1 h. Immediately after the heat curing process, the 400 µm‐high microstructures were removed. Subsequently, the mixture containing 10 wt.% CNTs was filled into the 400 µm microstructures and grooves, followed by vacuum degassing to eliminate entrapped air. After degassing, the assembly was moved to oven (80°C) for about four hours to ensure complete solidification. Finally, the conductive layer was gently peeled off from the template for subsequent performance testing.

### 4.4 Characterizations

Optical images were obtained using Carl Zeiss digital microscopy in conjunction with Olympus optical microscopy. The Keysight B2902A meter facilitated real‐time monitoring of electric current, supplying a voltage of 1 V. A high‐precision electronic balance (Lichen, YP200001D, China) recorded the exact force applied to the sensor, while the pressure was calculated by taking the ratio of the recorded force to the sensing area (1 cm × 1 cm). The elastic modulus was determined through precise displacement control using a Mark‐10 ESM303 Motorized Tension/Compression Test Stand, with real‐time pressure measurements captured by a Force Gauge (Model M5‐50). Voltage and current signals from the arrayed device were recorded with a Keysight DAQ970A data acquisition unit, which was equipped with a Keysight DAQM900A multiplexer module allowing for 40 parallel channels. The arrayed insole featured 10 interdigitated electrodes (with a finger width of 0.9 mm, gap width of 100 µm, and an area of 1 cm × 1 cm), while the arrayed dual‐factor authentication system included 12 interdigitated electrodes of the same specifications. Wearable signal acquisitions were performed by volunteers with ensured safety and informed written consent was obtained from participants before the demonstrations. In accordance with relevant regional regulations, the characterizations conducted in this study are in vitro, non‐clinical, and non‐toxic, thereby eliminating the need for formal approval from institutional authorities. Data analysis and processing were carried out using OriginLab software.

### 4.5 Simulation Model

Finite element analysis (FEA) was conducted in ABAQUS 2021 to investigate the mechanical response of the A_0.4_B_0.9_ conductive layer under variable conditions. A simplified two‐dimensional model was constructed, integrating key components, including a 200 µm‐thick flexible substrate, 4 micro‐dome pixels with 200 µm inter‐edge spacing, and a top electrode. The geometry was discretized using structured quadrilateral elements throughout. Within the conductive layer, pixels designated as A were assigned an elastic modulus of 2.07 MPa, while other regions exhibited 4.00 MPa elasticity. All components maintained a uniform Poisson's ratio of 0.2. To replicate physical interactions, contact was defined between the electrode's bottom surface and the conductive layer's upper surface. Self‐contact interactions were also defined within the conductive layer to improve simulation accuracy. Boundary constraints restricted motion at the substrate's bottom and lateral edges. Displacements of 150, 350, and 550 µm were sequentially applied to the electrode's top surface, enabling computation of the conductive layer's deformation profiles under each loading condition.

### 4.6 Working Principle of Morse Code Communication

The sensors collect pressure signals through an Analog‐to‐Digital Converter (ADC) module, performing the conversion from pressure signals to analog signals and subsequently to digital signals. These digital signals are then processed by a microcontroller unit (MCU, STM32F103C8T6), which then wirelessly transmits the data to a mobile terminal via an HC‐05 Bluetooth module. On the mobile terminal, a corresponding application (APP) receives the pressure intensity signals, processes the data, and visualizes the results accordingly.

### 4.7 Machine Learning

Within the overall system, the deep learning component is instantiated as a one‐dimensional classification network specifically tailored for handwritten digit recognition from current signals, termed the Hierarchical Stroke‐Sequence Network (HSS‐Net). The network takes as input raw temporal waveforms produced during the writing process and interprets them as sequences of latent ``stroke'' features. It then performs feature extraction, temporal modeling, and final classification in a cascaded manner. Formally, given an input signal tensor

$$X\in R^{B\times 1\times L_{\mathrm{in}}}$$

where *B* is the batch size and *L*_in_ is the variable sequence length, the output of HSS‐Net is defined as:

$$\;\hat{Y}=F_{\mathrm{cls}}\;\left(F_{\mathrm{seq}}\left(F_{\mathrm{ext}}\left(X\right)\right)\right)$$

where $F_{\mathrm{ext}}$, $F_{\mathrm{seq}}$, and $F_{\mathrm{cls}}$ denote the feature extraction module, the temporal sequence modeling module, and the classifier, respectively.

### 4.8 Hierarchical Waveform Feature Extraction Module

To automatically learn stroke‐level local patterns from raw one‐dimensional current signals, the feature extraction module $F_{\mathrm{ext}}$ is implemented as a deep one‐dimensional convolutional neural network based on a residual learning framework. The input *X* is first passed through an initial convolutional layer to expand the channel dimension. It is then processed by a stack of residual block groups, each comprising several residual blocks with skip connections to facilitate gradient flow and stabilize the training of deep architectures.

As depth increases, the number of feature channels is progressively enlarged, while temporal resolution is reduced using interleaved max‐pooling operations. This yields a multi‐scale, pyramid‐like representation of the input waveform that captures increasingly abstract semantic patterns.

Because the handwriting speed varies across samples, the original sequence length *L*_in_ is not fixed. To handle this, an adaptive average pooling layer is applied at the end of the CNN backbone to uniformly resample the temporal dimension to a fixed length *T*. The output of the feature extraction module is thus given by

$$H_{\mathrm{cnn}}\in R^{B\times C\times T}$$

where *C* is the final number of channels. This representation provides a unified‐length sequence of stroke‐like features for subsequent temporal modeling.

### 4.9 Composite Temporal Dependency Modeling Module

In the temporal modeling stage, HSS‐Net treats *H*_cnn_ as an ordered sequence of stroke features and explicitly models both local and global temporal dependencies. To retain information about relative temporal positions, a learnable positional encoding *P* is added to the CNN features:

$$\;H_{\mathrm{seq}}^{\left(0\right)}=H_{\mathrm{cnn}}+P$$

Next, $H_{\mathrm{seq}}^{\left(0\right)}$ is fed into a bidirectional long short‐term memory network (Bi‐LSTM), which encodes the sequence in both forward and backward directions and captures local to medium‐range temporal context:

$$\;H_{\mathrm{lstm}}=\mathrm{Bi}-\mathrm{LSTM}\left(H_{\mathrm{seq}}^{\left(0\right)}\right)$$

Although Bi‐LSTM is effective for modeling sequential data, it may still be limited in capturing very long‐range dependencies. To address this, we place a Transformer encoder layer with multi‐head self‐attention on top of the Bi‐LSTM outputs. The self‐attention mechanism allows the model to directly compute interactions between any pair of temporal positions, thereby modeling long‐range correlations and complex structural patterns in the digit‐writing process. The output of this stage is denoted by

$$H_{\mathrm{trans}}=\mathrm{TransformerEncoder}\left(H_{\mathrm{lstm}}\right)$$

### 4.10 Classification and Prediction Module

For classification, we first aggregate the temporal information in *H*_trans_ into a compact global representation by applying global average pooling over the temporal dimension. The resulting feature vector is then passed through a multilayer perceptron (MLP) equipped with regularization techniques such as batch normalization and dropout. Finally, a Softmax layer produces the probability distribution over the *K* digit classes:

$$\;\hat{Y}=\mathrm{Softmax}\left(\mathrm{MLP}\left(\mathrm{GlobalAvgPool}\left(H_{\mathrm{trans}}\right)\right)\right),\hat{Y}\in R^{B\times K}$$

### 4.11 Experimental Setup and Training Strategy

During training, the parameters of HSS‐Net are optimized by minimizing the standard categorical cross‐entropy (CE) loss between the predicted class probabilities $\hat{Y}$ and the one‐hot encoded ground‐truth labels $Y\in {\left\{0,1\right\}}^{B\times K}$. The loss over a mini‐batch is defined as:

$$\;L_{\mathrm{CE}}=-\frac{1}{B}\sum_{i=1}^{B}\sum_{k=1}^{K}y_{ik}\log{\hat{y}}_{ik}$$

where ${\hat{y}}_{ik}$ and ${\hat{y}}_{ik}$ denote the ground‐truth indicator and predicted probability for the *k*‐th class of the *i*‐th sample, respectively.

For all experiments, the dataset is split into training, validation, and test sets with a ratio of 7:2:1, ensuring reliable model selection and performance evaluation. The HSS‐Net model is trained on an NVIDIA Tesla A100 GPU. To improve robustness and mitigate the influence of random initialization and data shuffling, we train the model multiple times with different random seeds and report the average performance across runs. All results are computed based on 10‐times averaged metrics, providing a more stable and reproducible assessment of the proposed HSS‐Net within the overall system.

### 4.12 Design and Signal Transmission of Insole System

Data acquisition is achieved using the Keysight DAQ970A in combination with the multi‐channel acquisition board DAQM900A. During the data collection process, the acquisition board continuously switches channels to collect signals from the ten channels. The collected data is transmitted to a computer via a communication interface, where it is visualized using LabVIEW software. Ten Gaussian surfaces are generated based on the ten data points collected from the different sensor positions, with the physical location of each sensor corresponding to the center of its respective Gaussian surface. By superimposing these ten Gaussian surfaces and performing a point‐wise multiplication with a pre‐defined two‐dimensional array (the insole strength map) in LabVIEW, we obtain the final distribution diagram of heat map, which is displayed on the computer interface.

### 4.13 Dual‐Factor Authentication System

The dual‐factor authentication system was designed for password input and recognition. The data were collected by a Keysight DAQ970A in conjunction with a multi‐channel acquisition board DAQM900A. During the data acquisition process, the acquisition board continuously switches channels to sequentially collect signals from the 12 channels, transmitting the data to the customized LabView interface. Once the program receives the data, it visualizes the information accordingly. The human‐machine interface was built for user setting of the desired password combination. After setting the password, users can click “Set” to finalize the selection. If an error occurs, users can click “Reset” to reconfigure the password.

## Author Contributions

**B.Z**. acquired funding, guided the whole project, reviewed and edited the paper. **Y.L**. participated in the characterization and data collection. **X.X**. achieved the data processing for machine learning. **Z.Y**. and **F.H**. contributed to the circuit design. **Y.L**. and **B.Q**. participated in data analysis and physiological signal acquisition. **R.L**. and **Z.D**. contributed to model simulation and experimental methodology. **J.L**. contributed to supervision and resources. All authors discussed the results and commented on the manuscript.

## Conflicts of Interest

The authors declare no conflicts of interest.

## Supporting information




**Supporting File 1**: advs76572‐sup‐0001‐SuppMat.docx.

**Supporting File 1**: advs76572‐sup‐0002‐VideoS1.mp4.

**Supporting File 3**: advs76572‐sup‐0003‐VideoS2.mp4.

**Supporting File 4**: advs76572‐sup‐0004‐VideoS3.mp4.

**Supporting File 5**: advs76572‐sup‐0005‐VideoS4.mp4.

**Supporting File 6**: advs76572‐sup‐0006‐VideoS5.mp4.

**Supporting File 7**: advs76572‐sup‐0007‐VideoS6.mp4.

**Supporting File 8**: advs76572‐sup‐0008‐VideoS7.mp4.

**Supporting File 9**: advs76572‐sup‐0009‐VideoS8.mp4.

## Data Availability

The data that support the findings of this study are available from the corresponding author upon reasonable request.
