# Supplementary material for: Strategy of Triple‐Gradient in Binary Pixels for Flexible Pressure Sensing with High Sensitivity and Wide‐Range Linearity
Source: Adv Sci (Weinh). 2026 Jul 14:e76572. Online ahead of print. doi: 10.1002/advs.76572 (PMC13367112; doi:10.1002/advs.76572)
Supplement: Supplementary file 1 — Supporting File 1: advs76572‐sup‐0001‐SuppMat.docx. [file ADVS-9999-e76572-s005.docx]

**Supplementary Information**

**Strategy of Triple-gradient in Binary Pixels for Flexible Pressure Sensing with High Sensitivity and Wide-Range Linearity**

*Yifan Liu,^1^ Xiao Xu,^2^ Fengming Hu,^1,3^ Yuanzhe Liang,^1^ Zidong Yin,^1^ Biao Qi,^1^ Ruolin Liu^4,*^ Ziyi Dai,^5^ Jianyi Luo,^3^ and Bingpu Zhou^1,*^*

^1^Joint Key Laboratory of the Ministry of Education, Institute of Applied Physics and Materials Engineering, University of Macau, Avenida da Universidade, Taipa, Macau 999078, China

^2^School of Biomedical Engineering and Informatics, Nanjing Medical University, Nanjing 211166, China

^3^Research Center of Flexible Sensing Materials and Devices, School of Applied Physics and Materials, Wuyi University, Jiangmen 529020, China

^4^Shanghai Key Lab. of D&A for Metal-Functional Materials, School of Materials Science & Engineering, Tongji University, Shanghai 201804, China

^5^School of Integrated Circuits, Shandong University, Jinan, 250101 China

***Corresponding Author.**

Ruolin Liu, Email: liuruolin00@tongji.edu.cn

Bingpu Zhou, Email: bpzhou@um.edu.mo. Fax: +853-88222426. Tel: +853-88224196.

Keywords: wearable sensors, binary pixel, gradient design, machine learning, human-machine interactions

**Contents**

[**Supplementary Video Captions. 3**](#_Toc220502870)

[**Supplementary Figures and Tables. 4**](#_Toc220502871)

[**Supplementary References 33**](#_Toc220502872)

Supplementary Video Captions.

**Video S1.** Real-time recording of Morse code “UNIVERSITY—OF—MACAU” generated by the customized circuit and interactive interface. The video was processed with 4X speed for play.

**Video S2.** Real-time mobile screen recording of Morse code “UNIVERSITY—OF—MACAU”. The video was processed with 4X speed for play.

**Video S3.** Real-time monitoring of toe standing, genu varum (bow-legged), normal standing, genu valgum (knock-kneed), heel contact, and flat feet. The video was processed with 3X speed for play.

**Video S4.** Real-time computer screen recording of toe standing, genu varum (bow-legged), normal standing, genu valgum (knock-kneed), heel contact, and flat feet. The video was processed with 3X speed for play.

**Video S5.** Real-time recording of the password-setting process.

**Video S6.** Real-time recording of successful password unlocking. The video was processed with 2X speed for play.

**Video S7.** Real-time recording of failed unlocking with incorrect password (correct digits, wrong intensity). The video was processed with 2X speed for play.

**Video S8.** Real-time recording of failed unlocking with incorrect password (correct intensity, wrong digits). The video was processed with 2X speed for play.

Supplementary Figures and Tables.

**
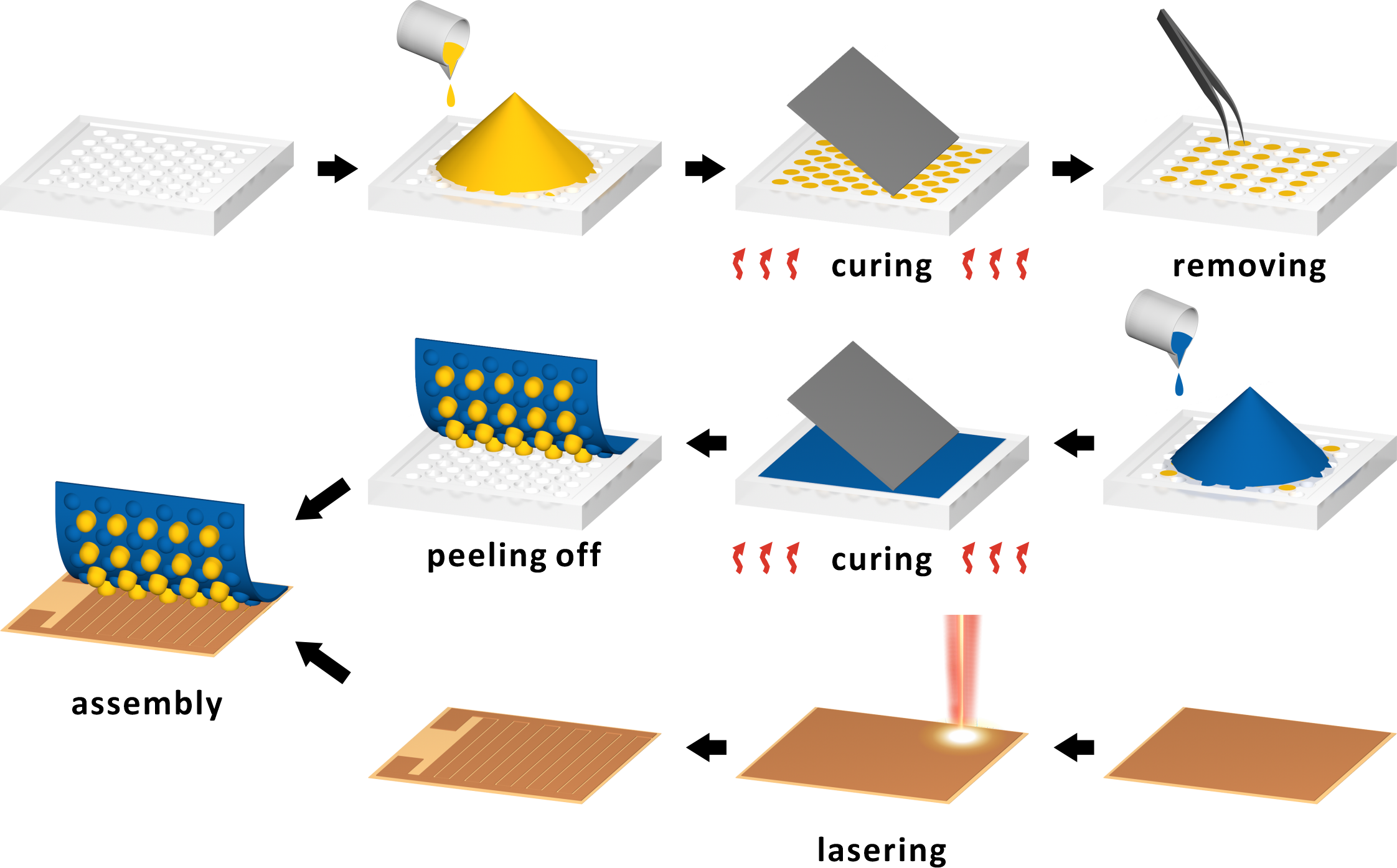
**

**Figure S1.** Schematic diagram of the fabrication process for the flexible resistive sensing layer. Here, the plastic template was prepared by standard engraving process, the interdigital electrodes were prepared by laser processing.

**
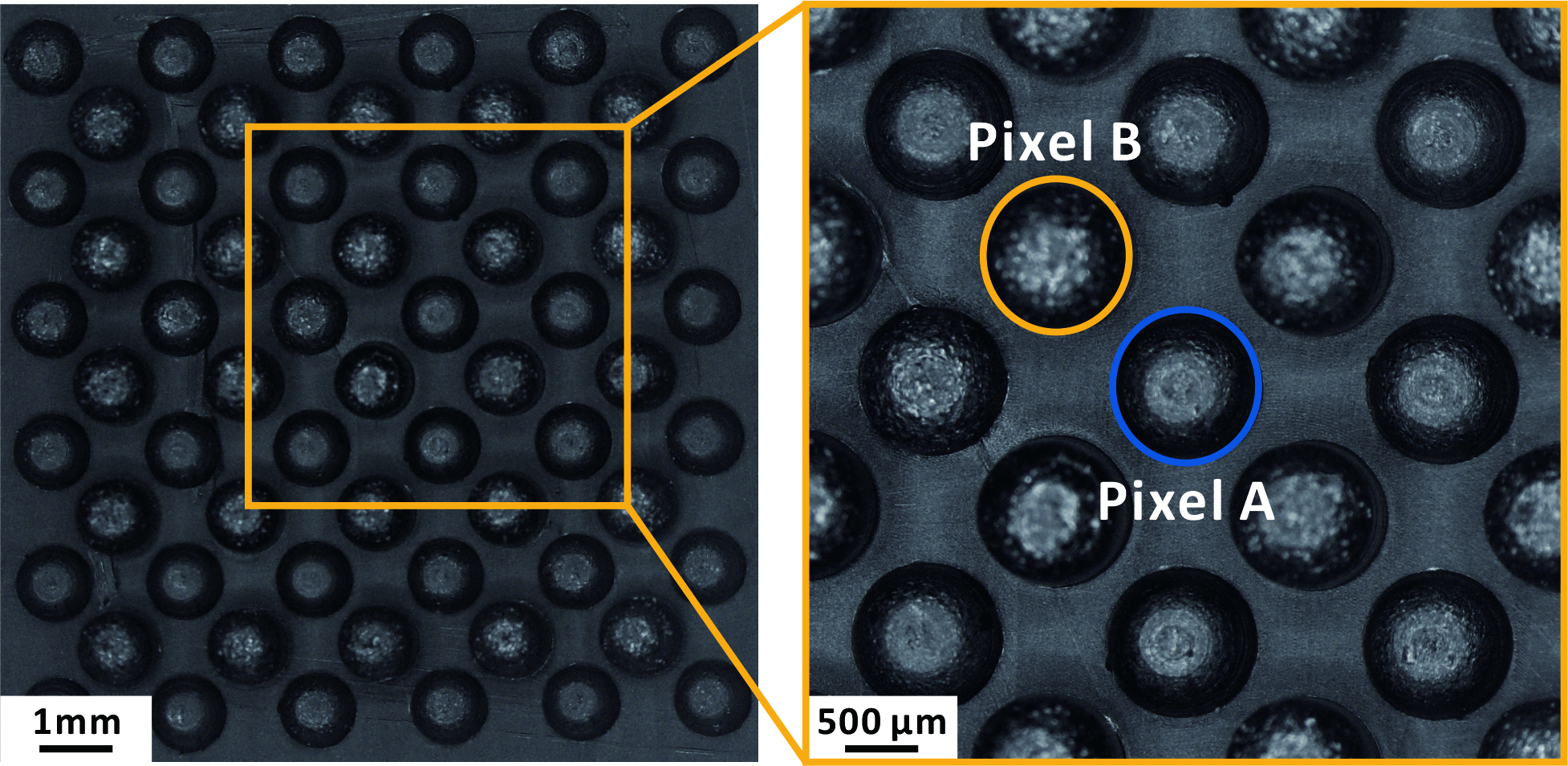
**

**Figure S2**. Optical images in top-view of the proposed resistive sensor with binary pixel design (Pixel A and Pixel B).


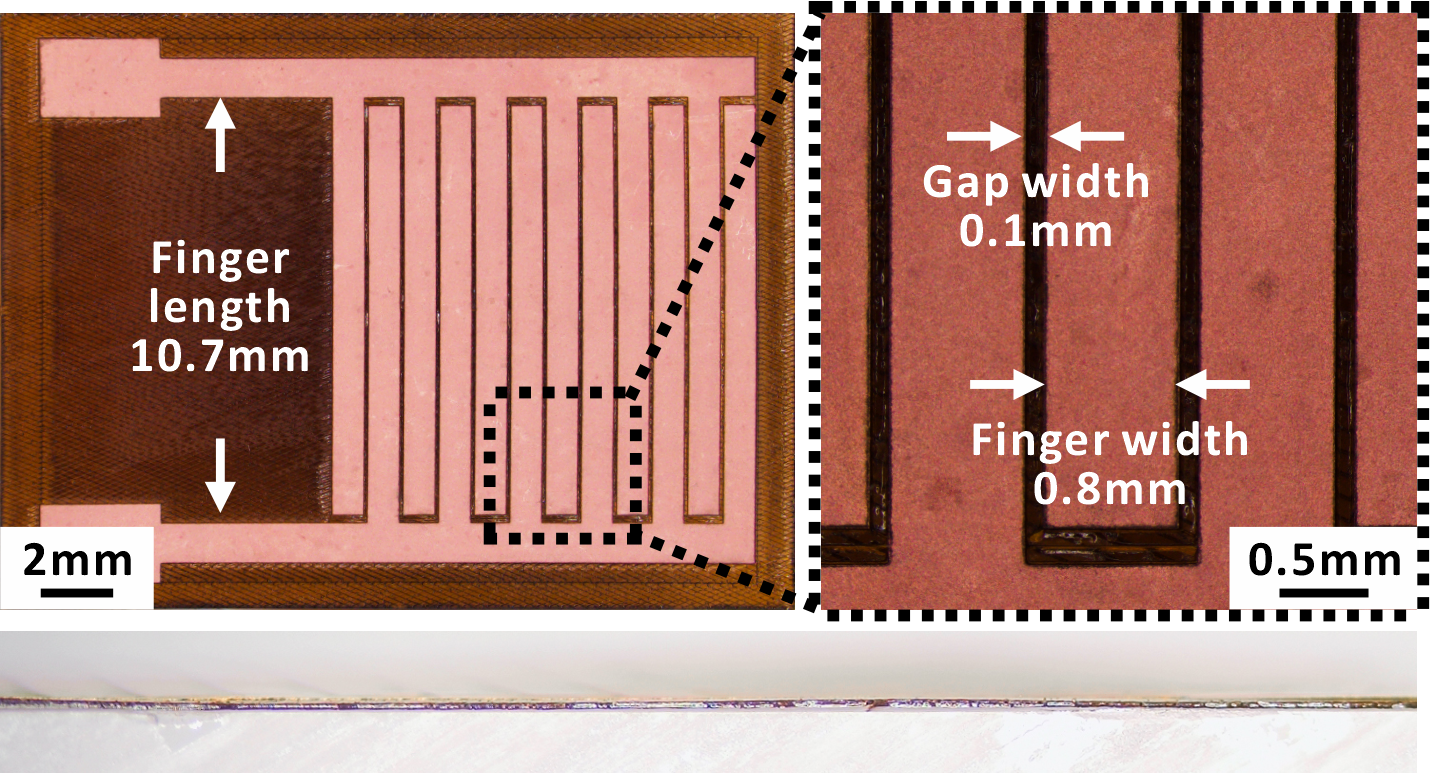


**Figure S3.** Optical images of the surface, gap width and finger width of the interdigitated electrodes.


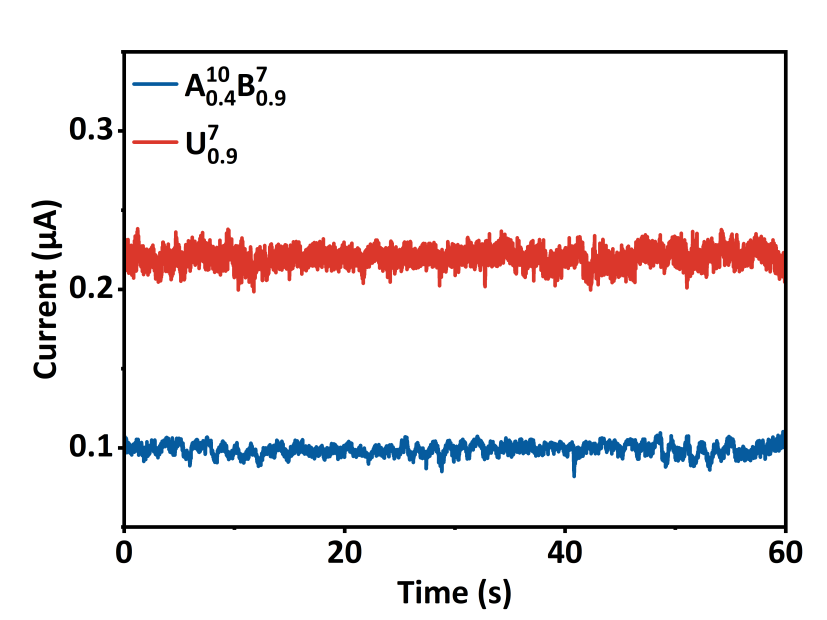


**Figure S4.** The stability of the initial current of A10 0.4B7 0.9 and U7 0.9 at the initial state.


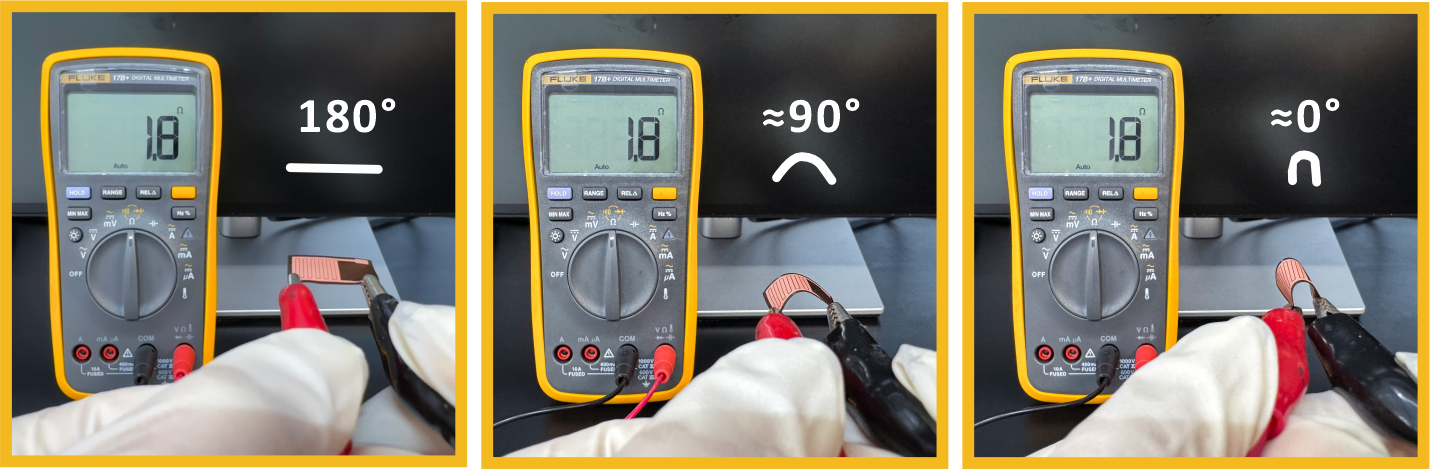


**Figure S5.** Resistance stability of the interdigitated electrodes at different bending degrees from 0° to 180°. The resistance values are measured by multimeter, indicating the electrode stability when exposed to mechanical deformations.


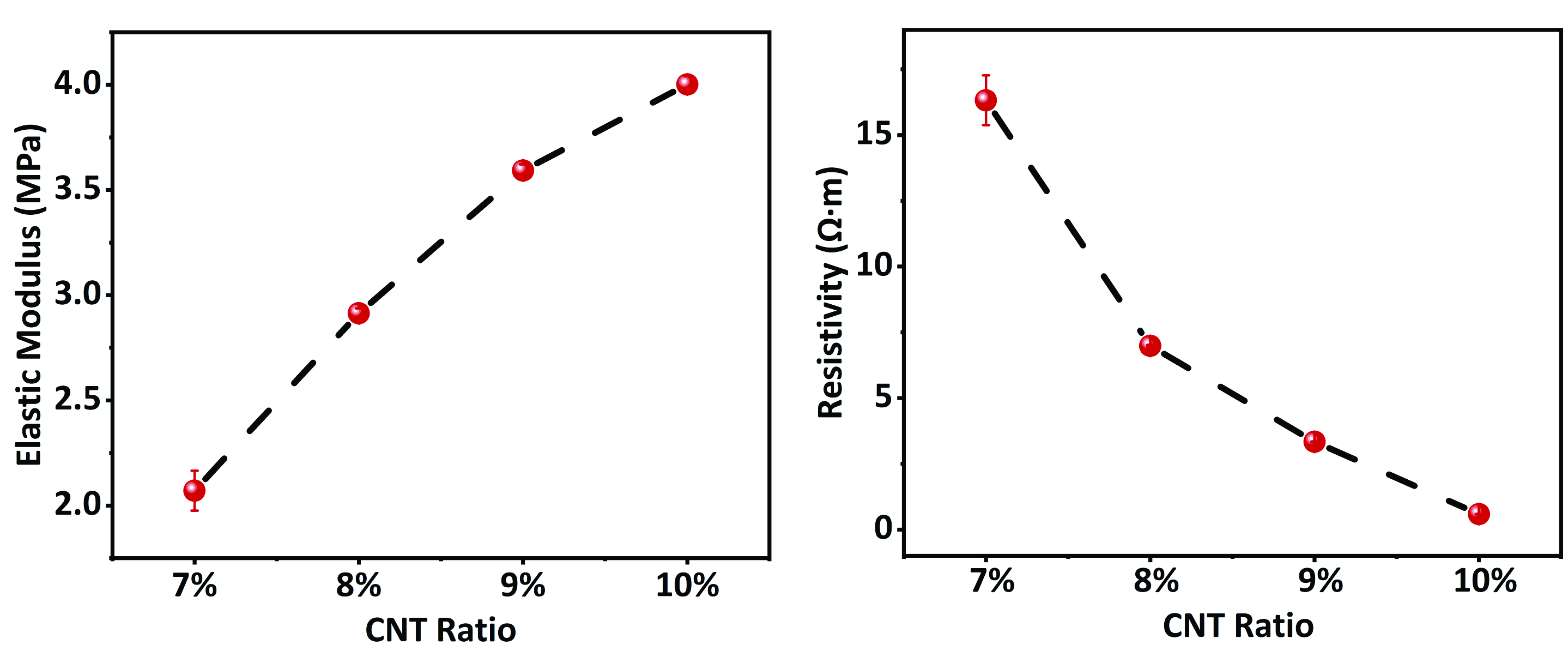


**Figure S6.** Variation trend of elastic modulus and resistivity (conductivity) when CNT mass ratio was changed from 7% to 10%.

**
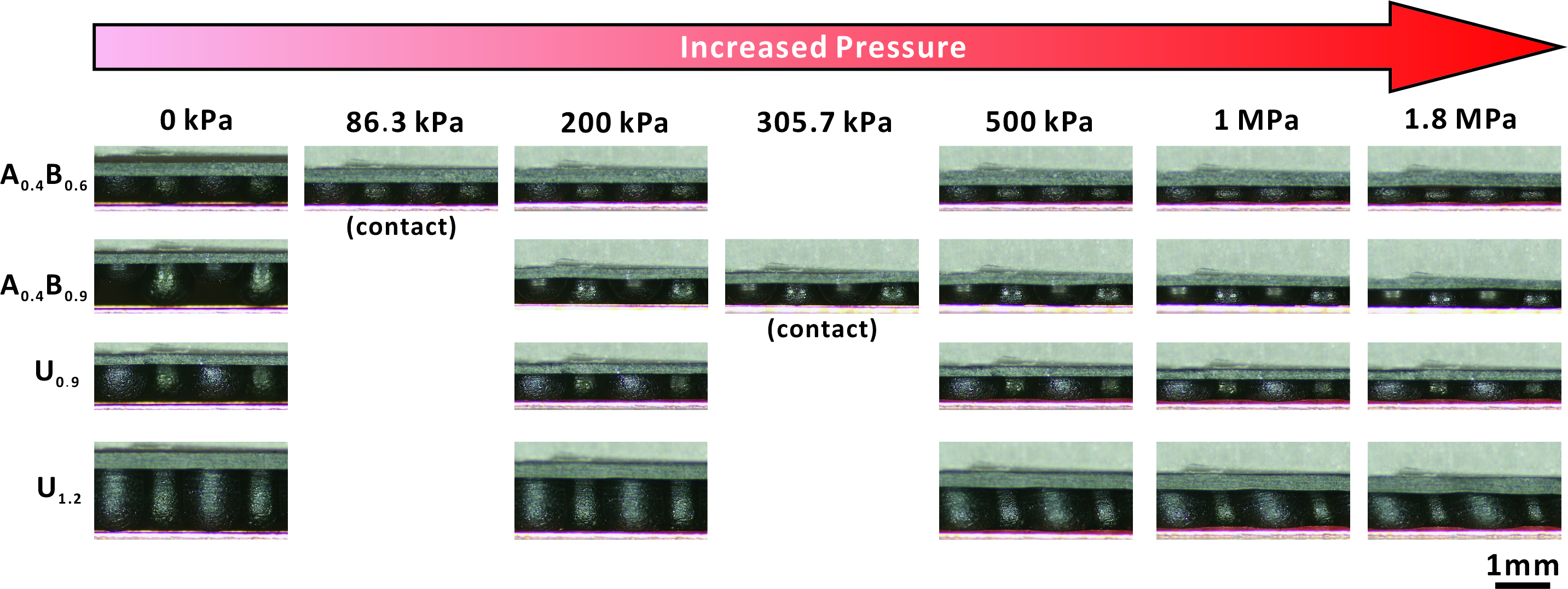
**

**Figure S7.** Optical images of A_0.4_ B_0.6_, A_0.4_ B_0.9_, U_0.9_ and U_1.2_ when exposed to different normal pressures up to 1.8 MPa.


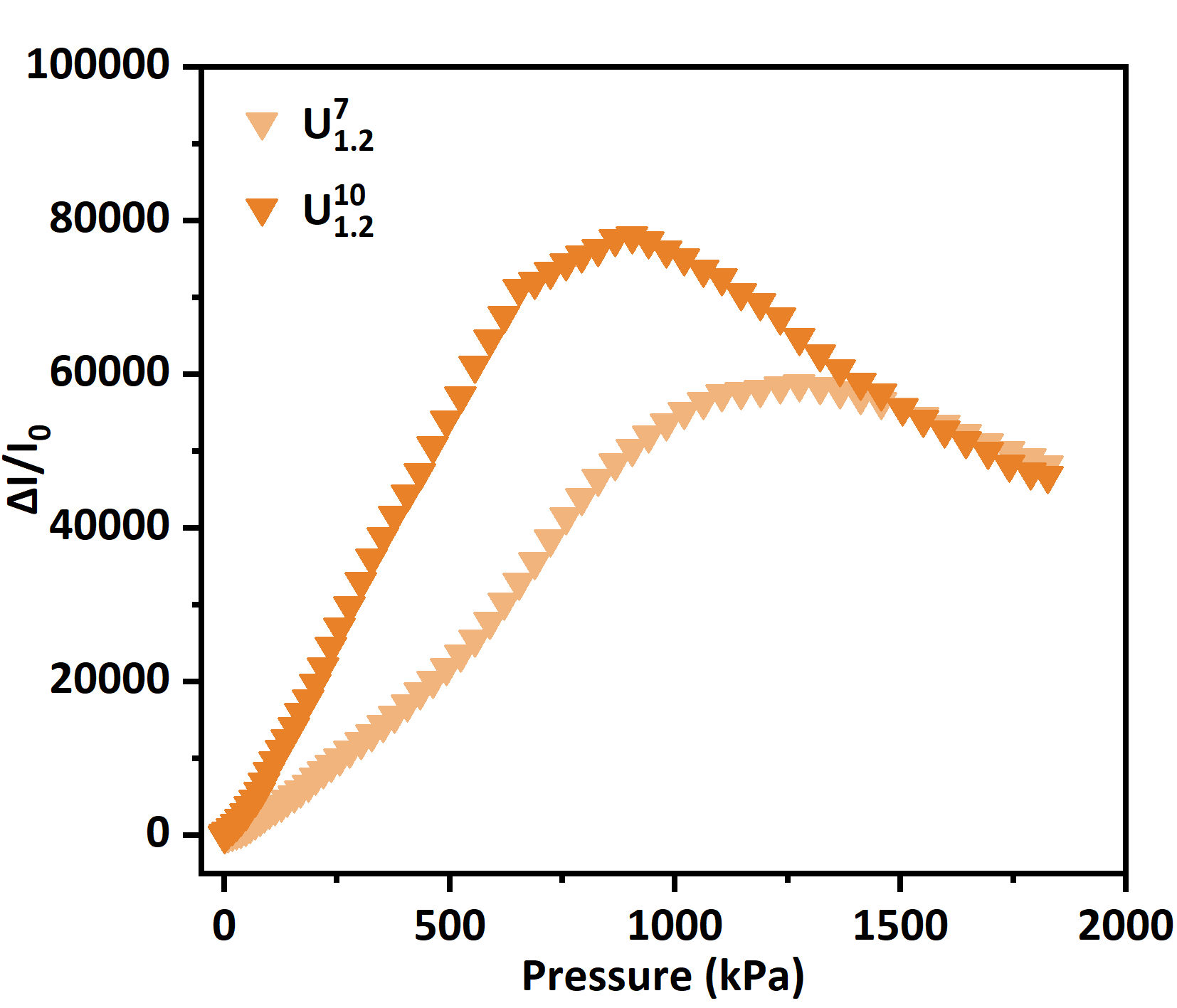


**Figure S8.** Relative resistiveness variation of U**7 1.2** and U10 1.2.

**
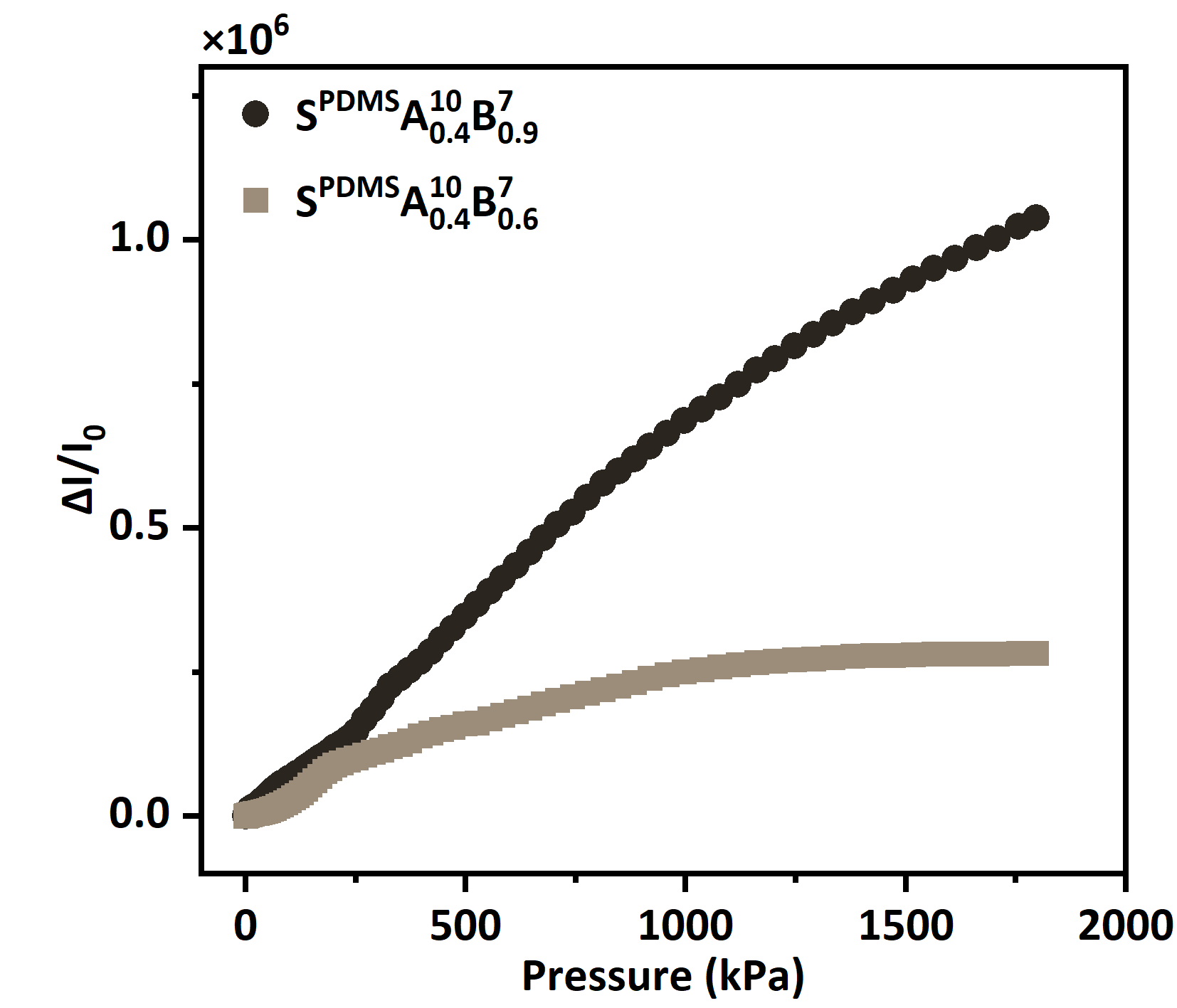
**

**Figure S9.** Relative resistiveness variation of S^PDMS^A10 0.4B7 0.9 and S^PDMS^A10 0.4B7 0.6. Here, the term of S^PDMS^ indicates the substrate is prepared by pure PDMS, without incorporation of conductive CNT/PDMS composites.


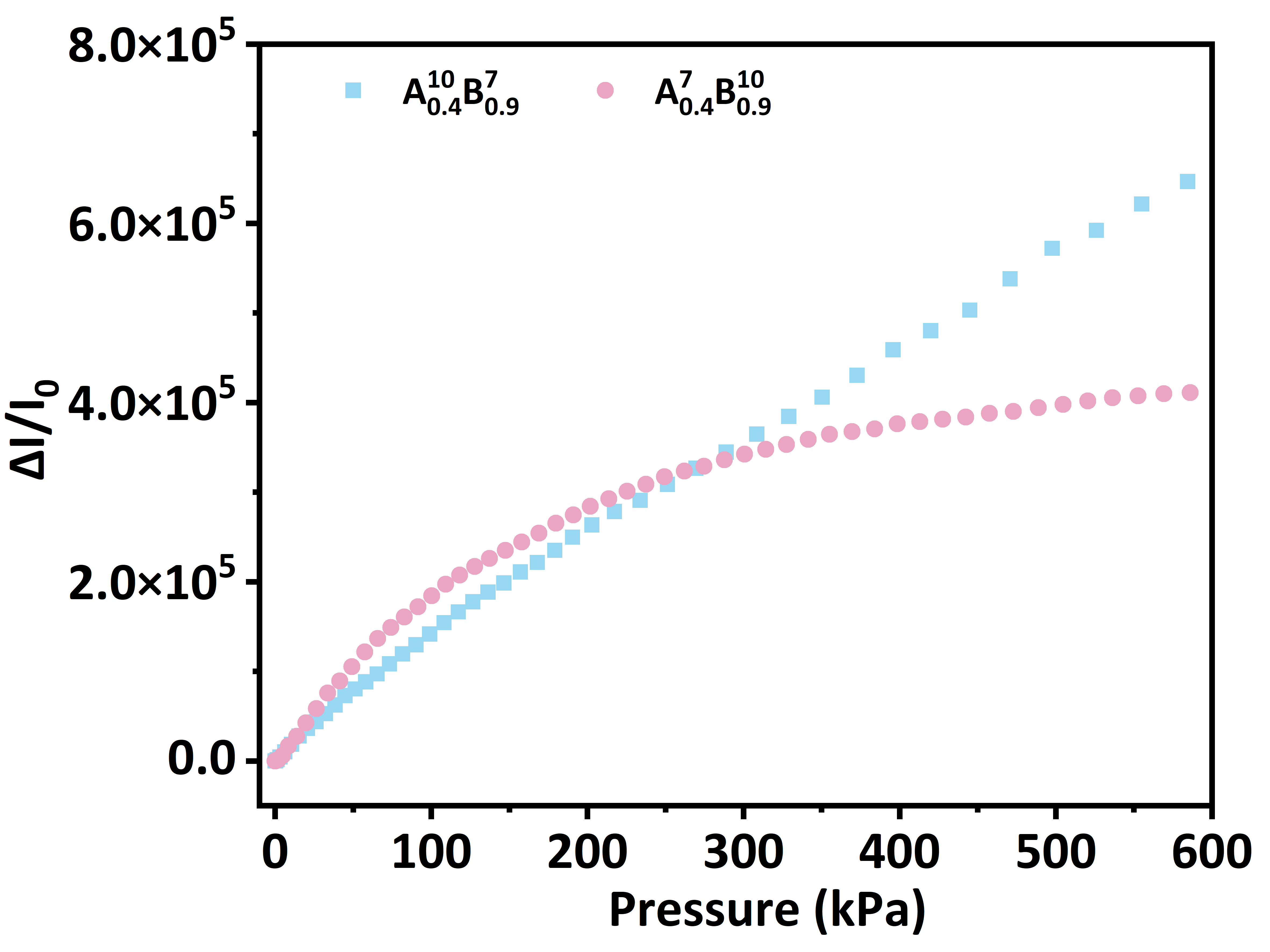


**Figure S10.** Relative current change in the gradient-based sensors A10 0.4B7 0.9 and A7 0.4B10 0.9.


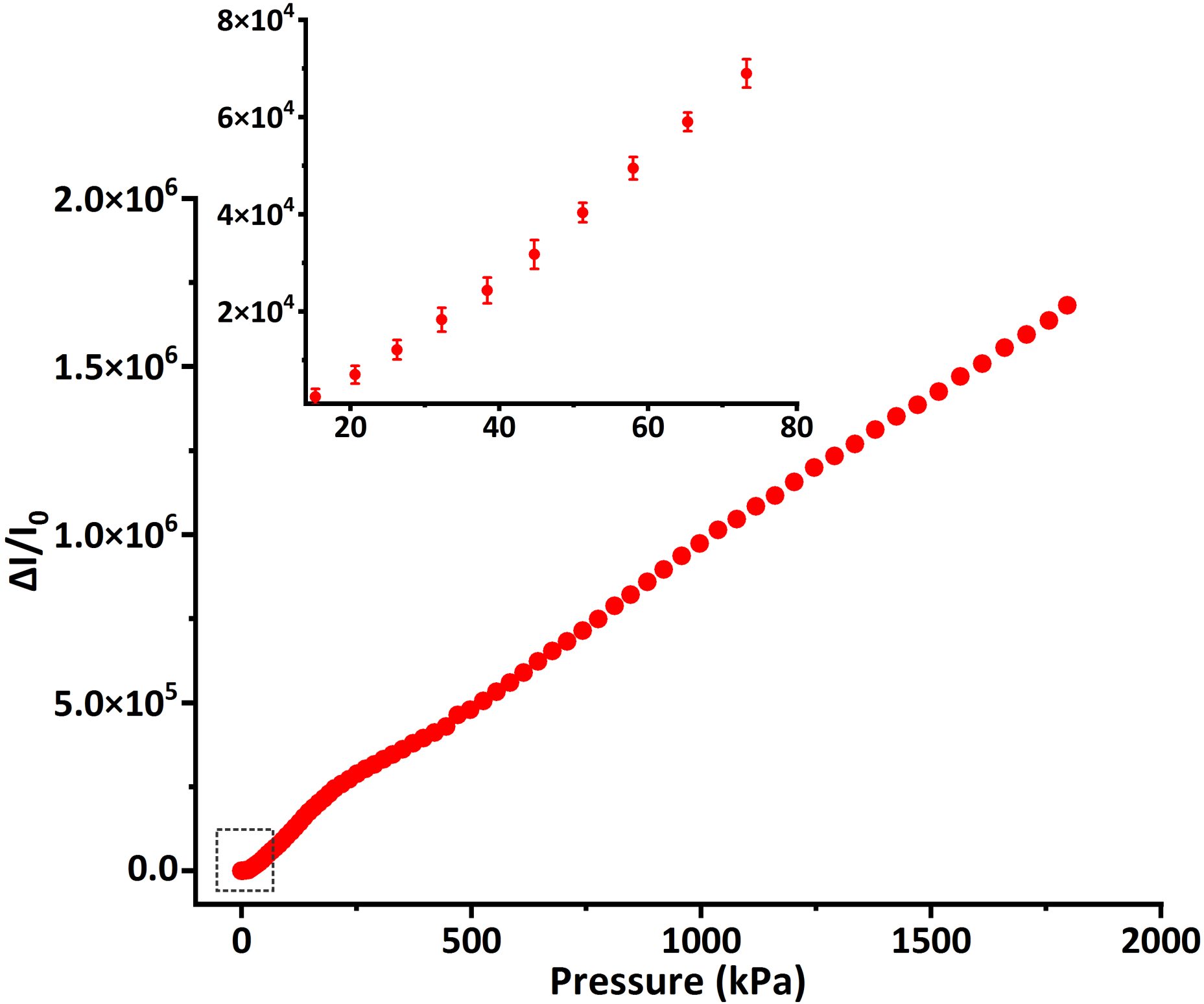


**Figure S11.** Relative current variation of A10 0.4B7 0.9 with error bars.


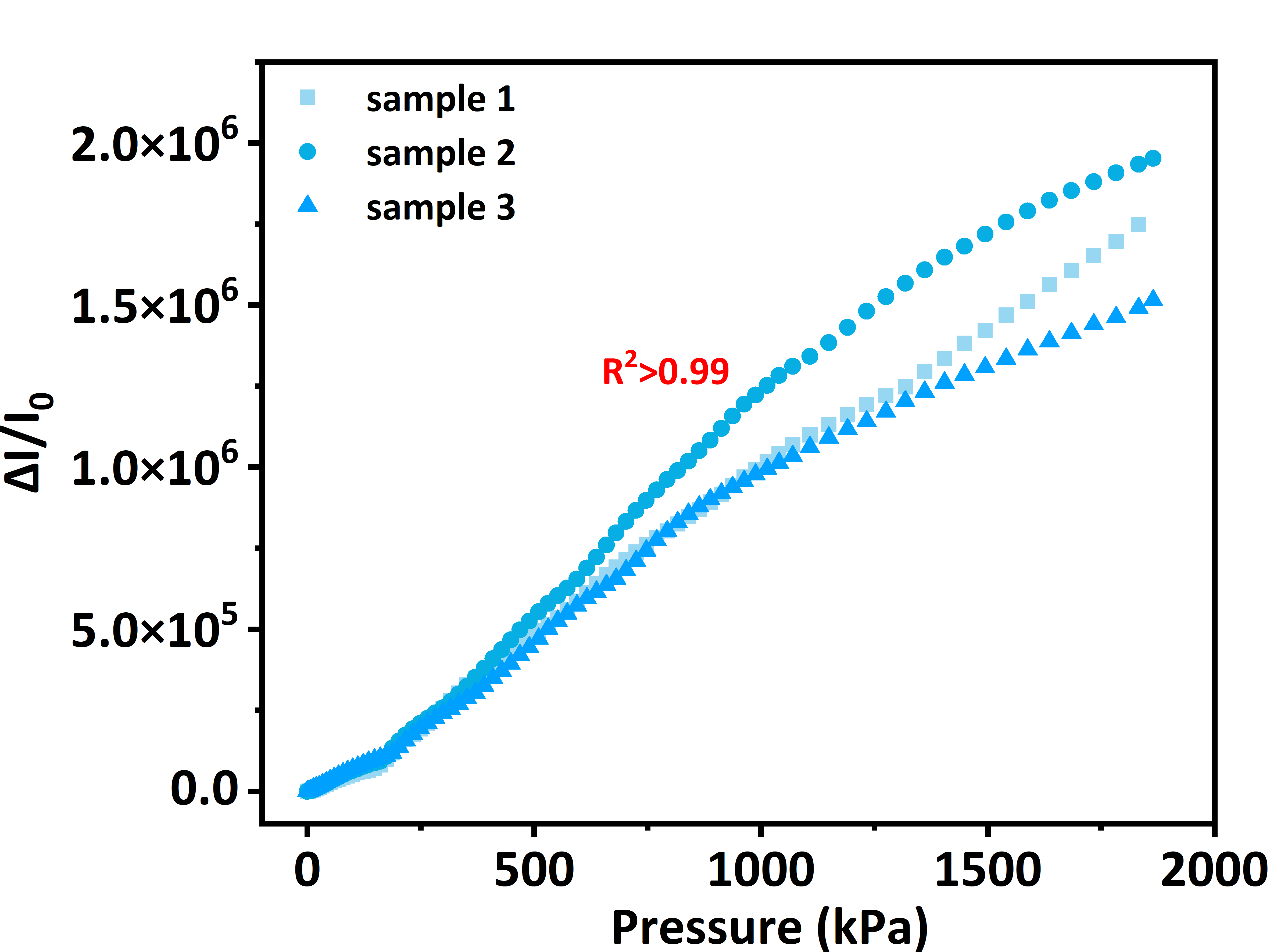


**Figure S12.** Relative current variation of A₀.₄B₀.₇ from different samples.


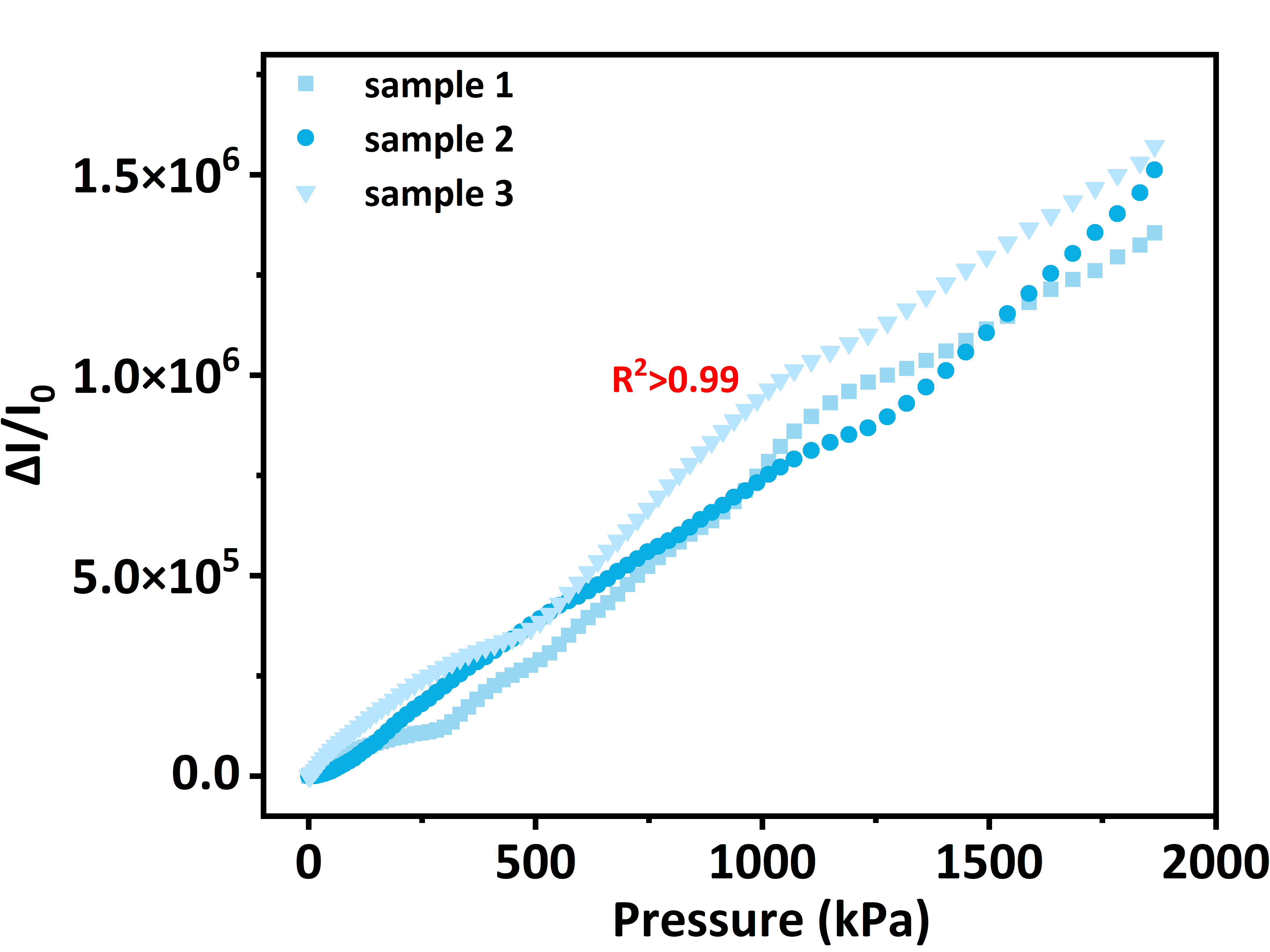


**Figure S13.** Relative current variation of A₀.₄B₀.₈ from different samples.


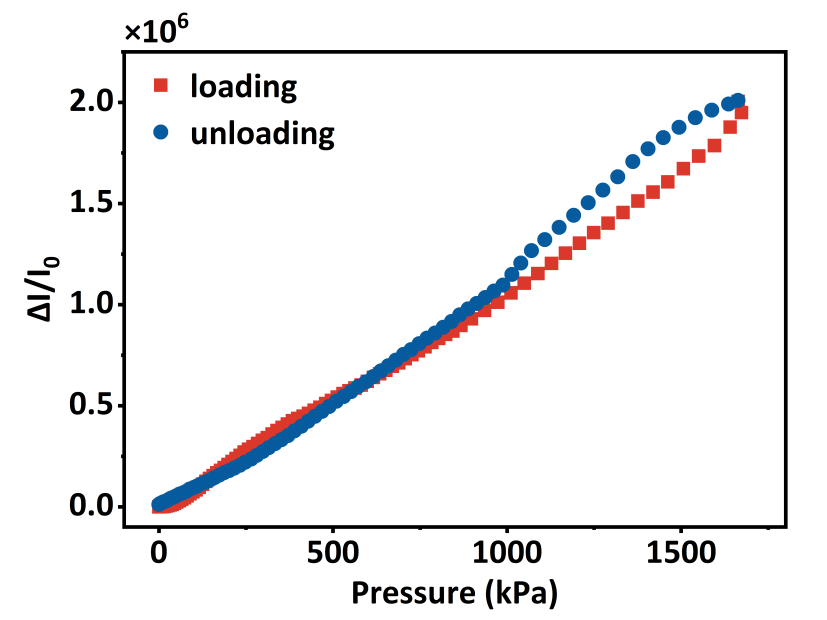


**Figure S14.** Dynamic response on the continuous loading/unloading pressure within the pressure range of 0~1800 kPa.


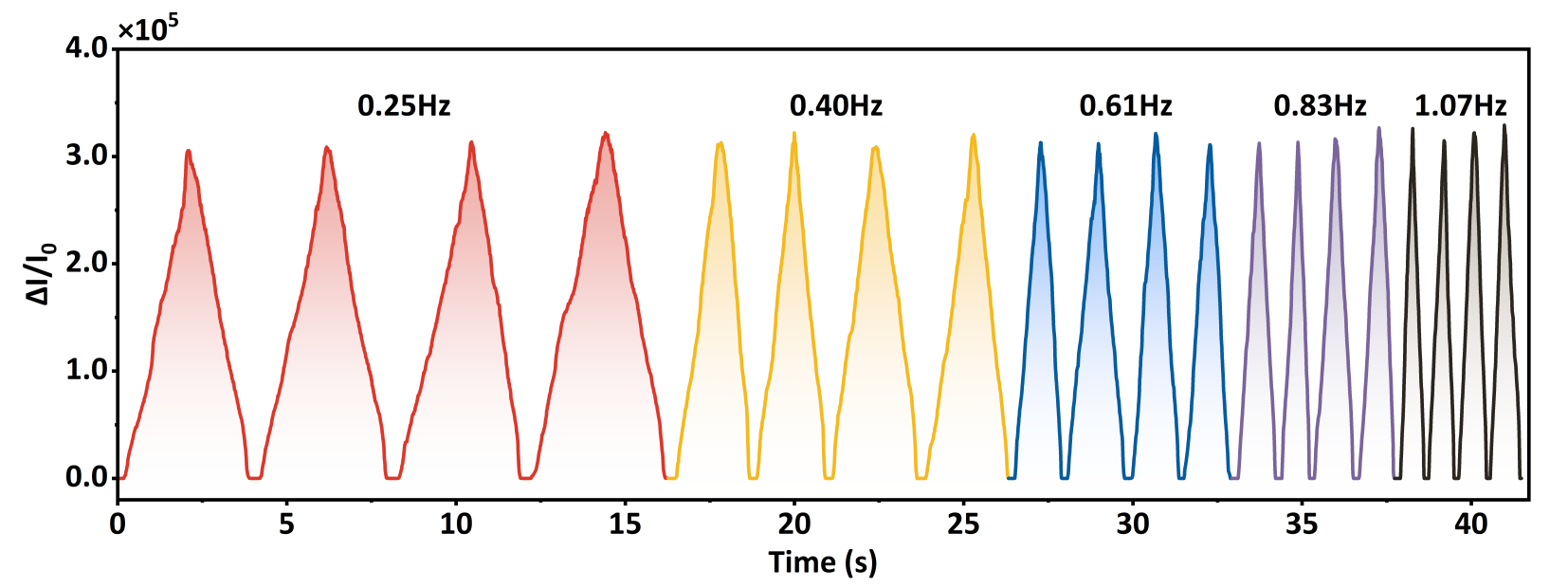


**Figure S15.** Relative current variation of flexible pressure sensors based on different pressing frequencies under the same normal pressure.


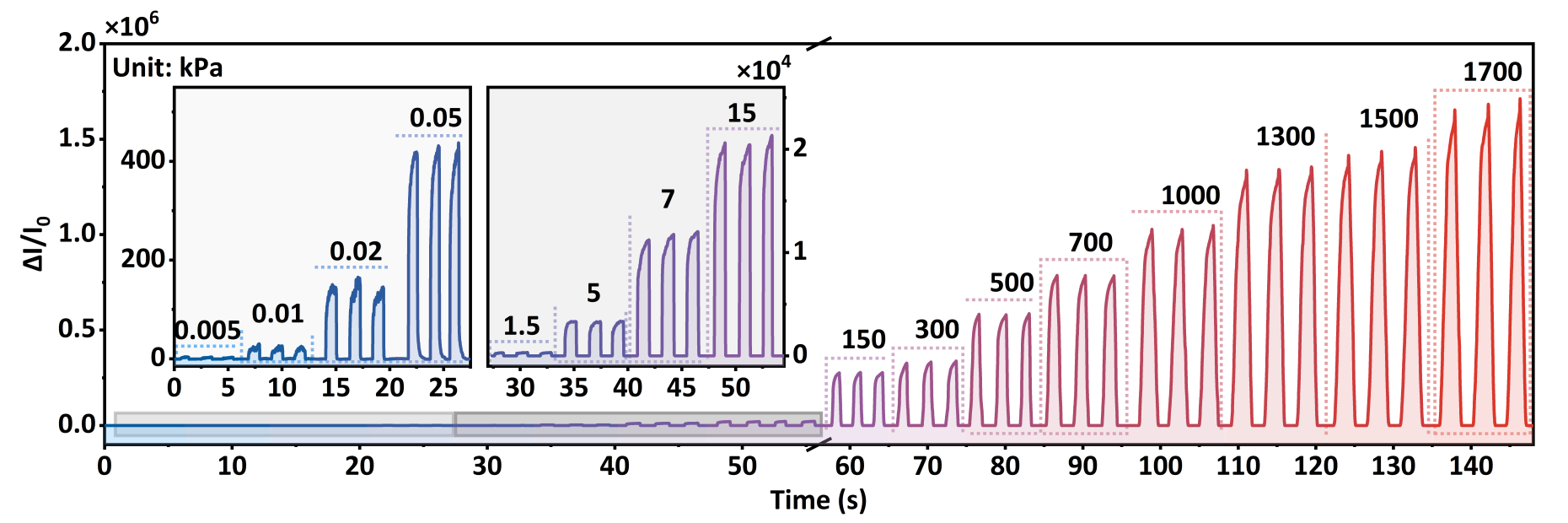


**Figure S16.** Dynamic stability of the sensor under pressure applications from 5 Pa to 1700 kPa.


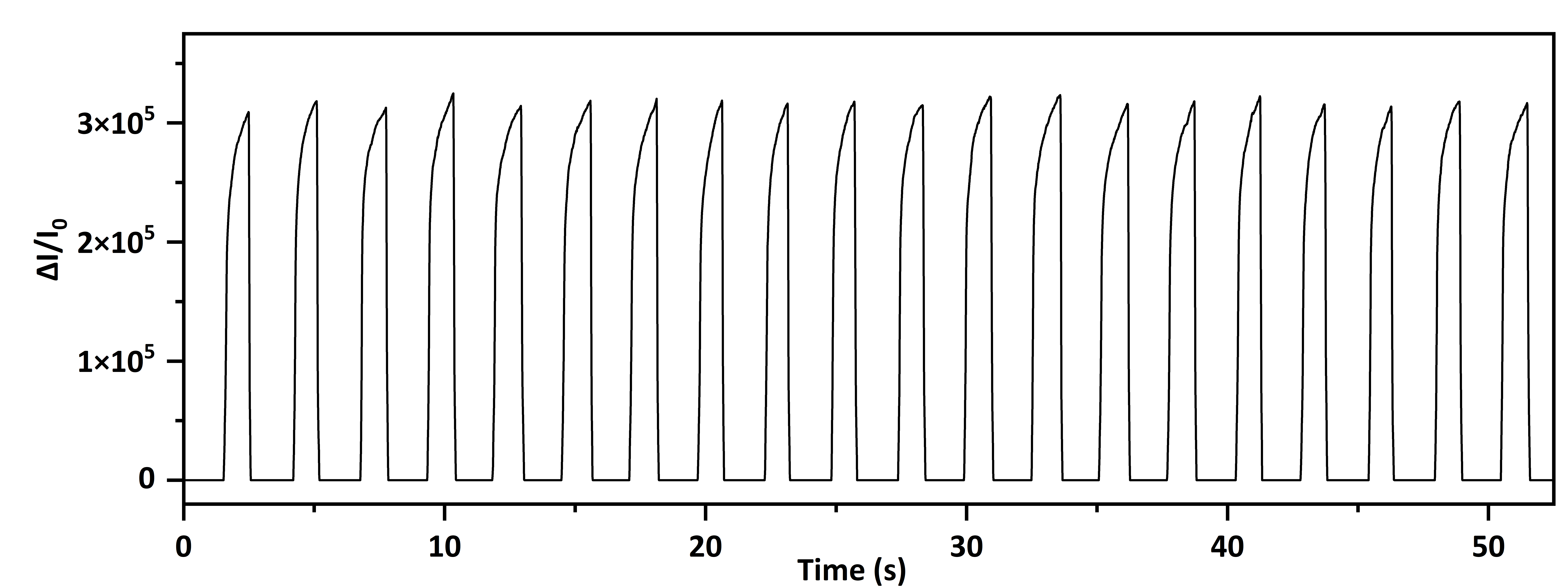


**Figure S17.** Current variation of the sensor after fatigue test when exposed to the same periodical pressure.


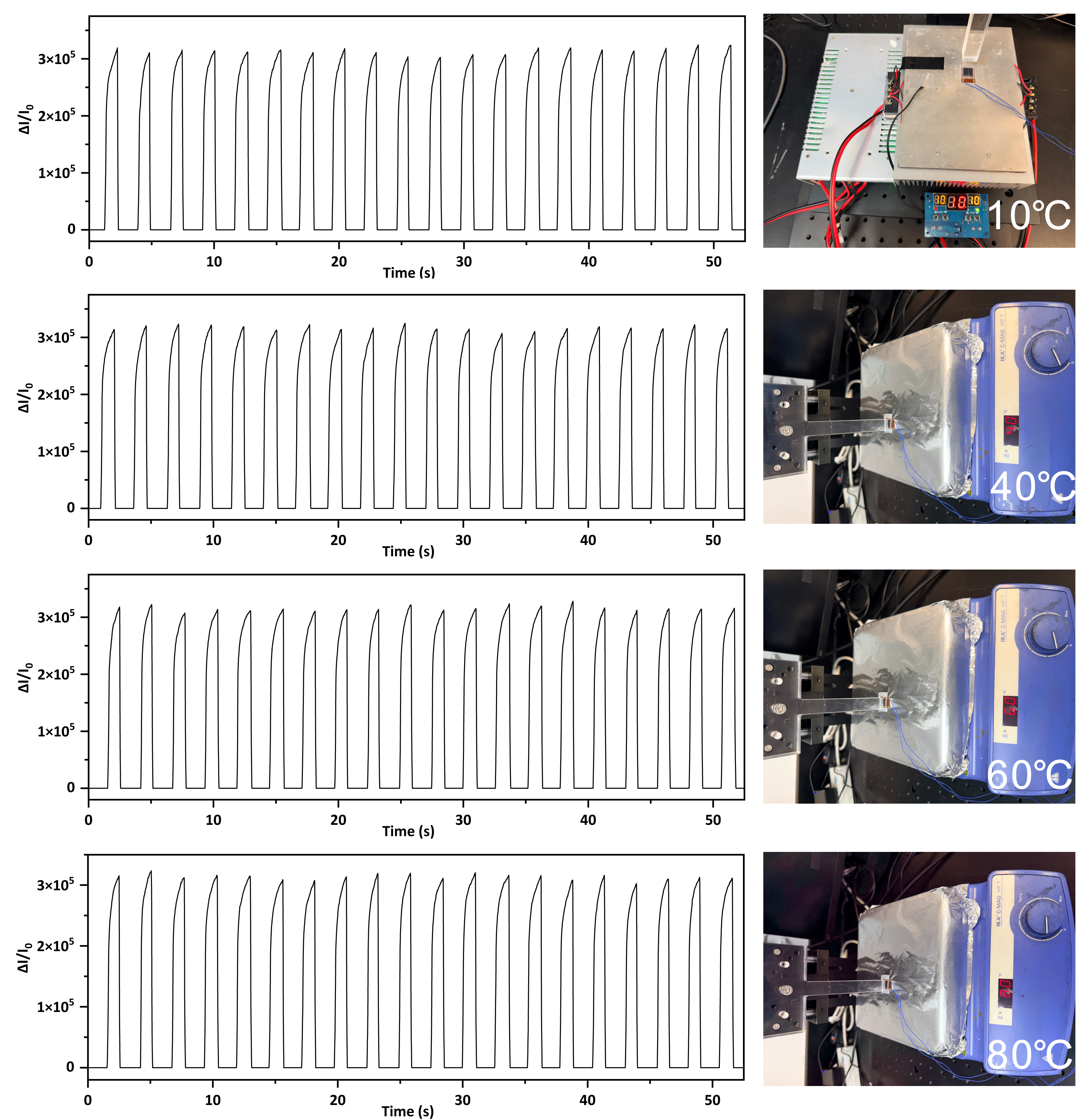


**Figure S18.** Stability of the sensor under a periodic pressure of ∼350 kPa at a frequency of 0.5 Hz under different environmental temperatures.


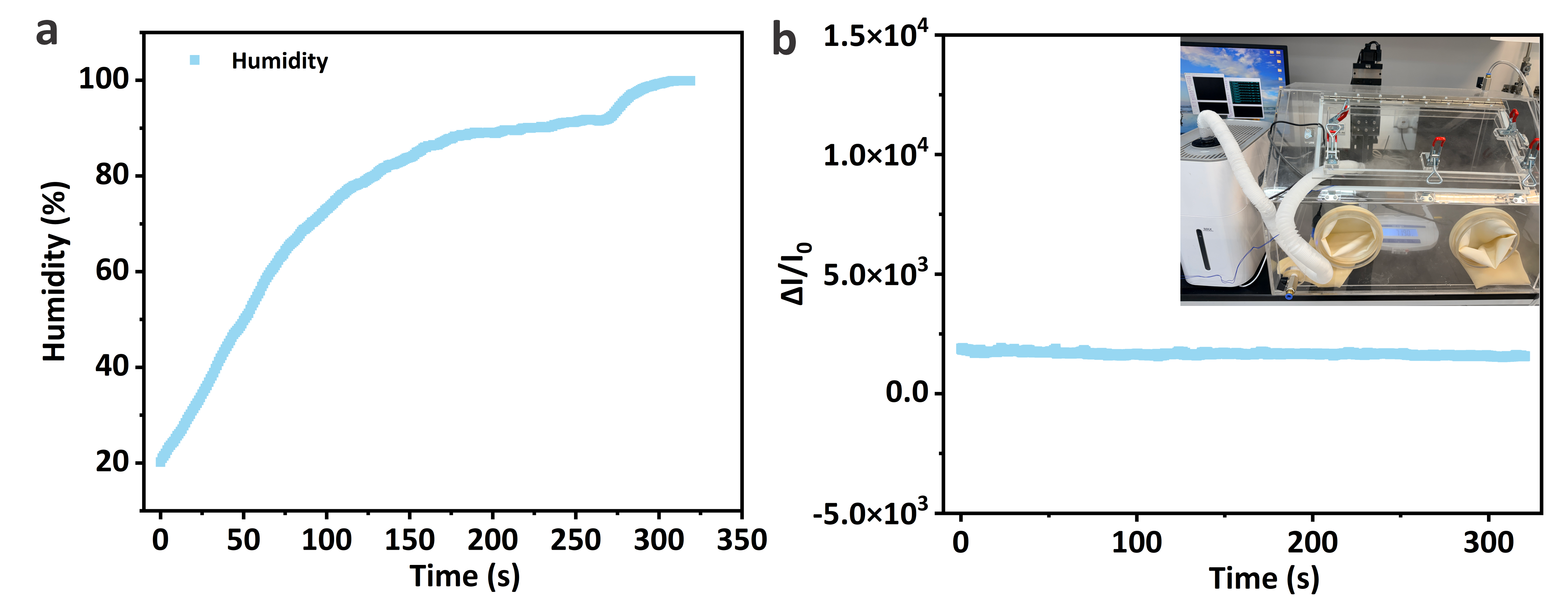


**Figure S19.** (a) Record of relative humidity variations from 20% to 99.9%. (b) Real-time monitoring of electrical currents under varying humidity conditions.


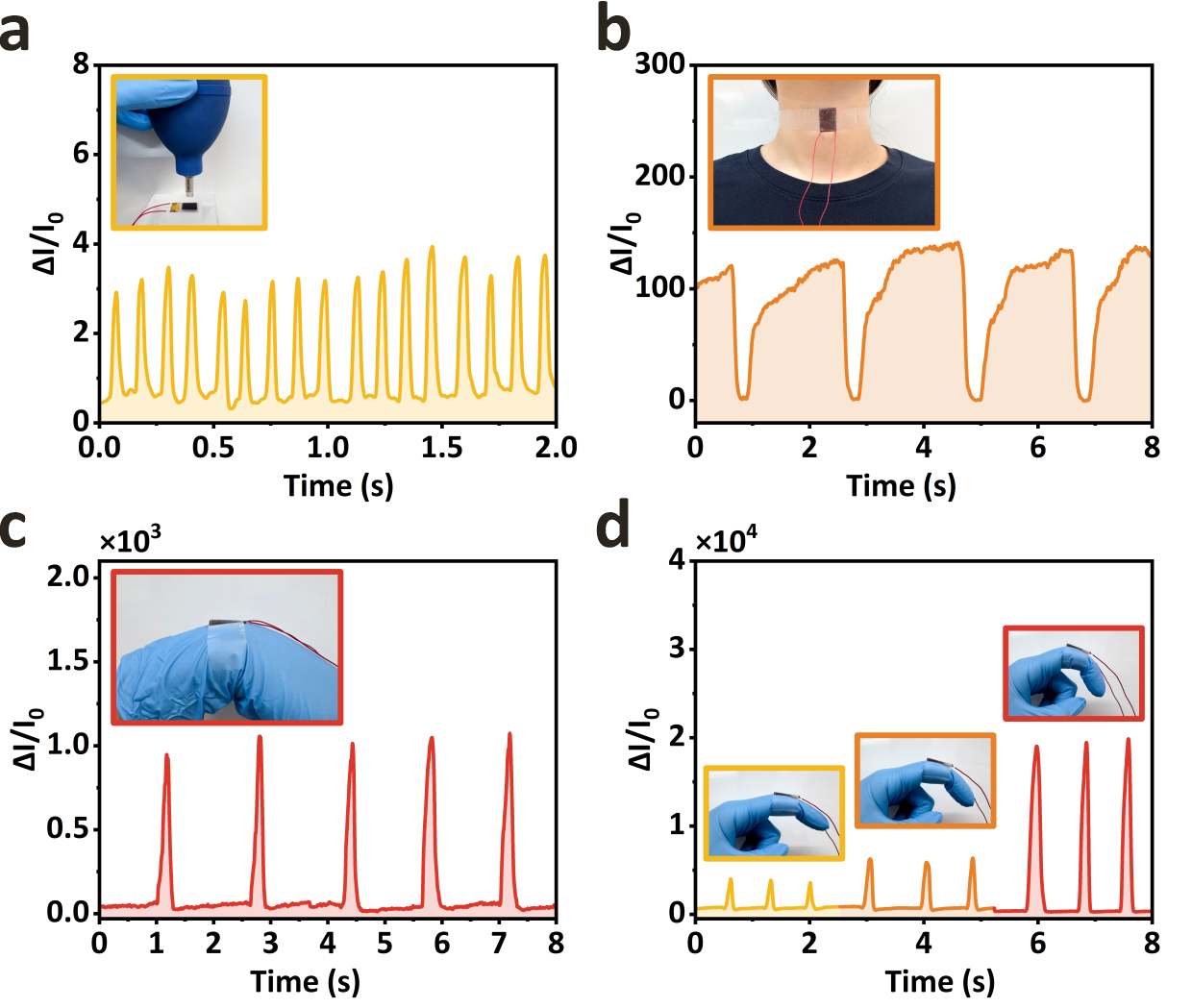


**Figure S20.** (a) Real-time monitoring of the air flow. (b) Real-time monitoring of swallowing motion. (c) Real-time response signals corresponding to the wrist flexion. (d) Real-time response signals corresponding to different angles of knuckle flexion.


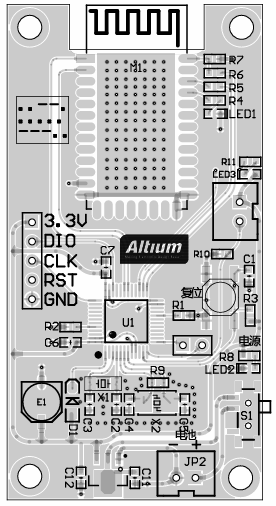


**Figure S21.** Optical images of the circuit boards used in this work for Morse code generation. This circuit enables Morse code generation based on resistive pressure detection. When pressure is applied to the sample, its resistance changes, and the STM32F103C8 microcontroller reads the corresponding voltage signal to determine the pressure level: low pressure (<150 kPa) produces a "dot", high pressure (150–1800 kPa) produces a "dash", and the absence of pressure for 3 seconds triggers a space symbol. These encoded signals are transmitted via the HC-05 Bluetooth module to a mobile device, where they are decoded and displayed as textual information. This design allows continuous reception and translation of multiple Morse code sequences.


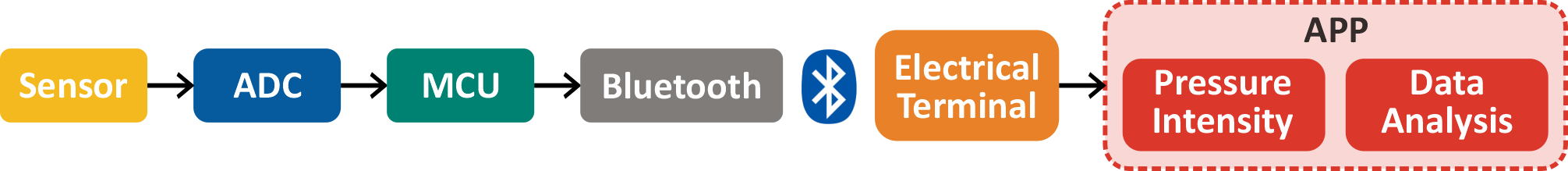


**Figure S22.** System architecture of the Morse code communication system.


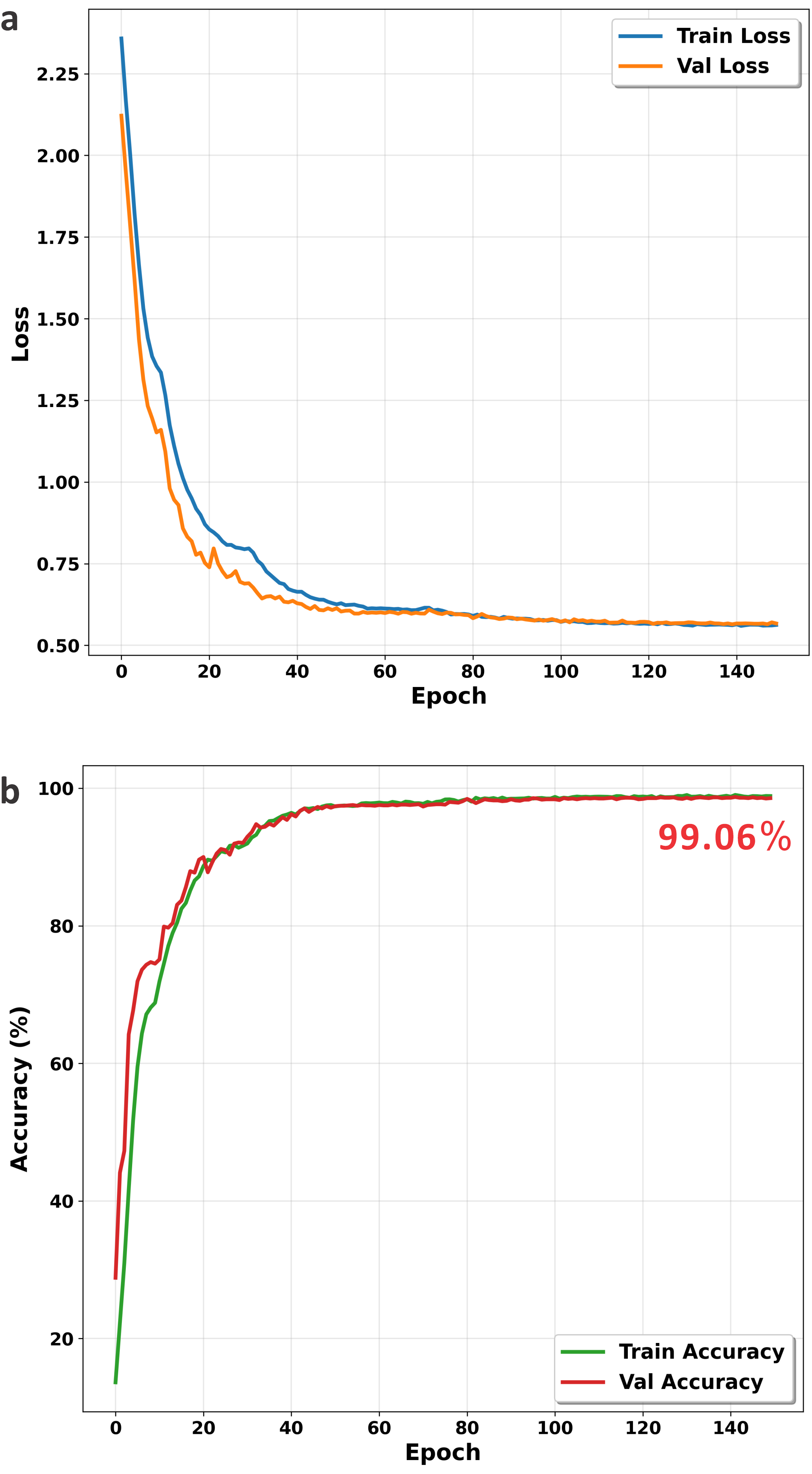


**Figure S23.** (a) Train and validation loss of epochs in the model. (b) Validation accuracy of epochs in the model.


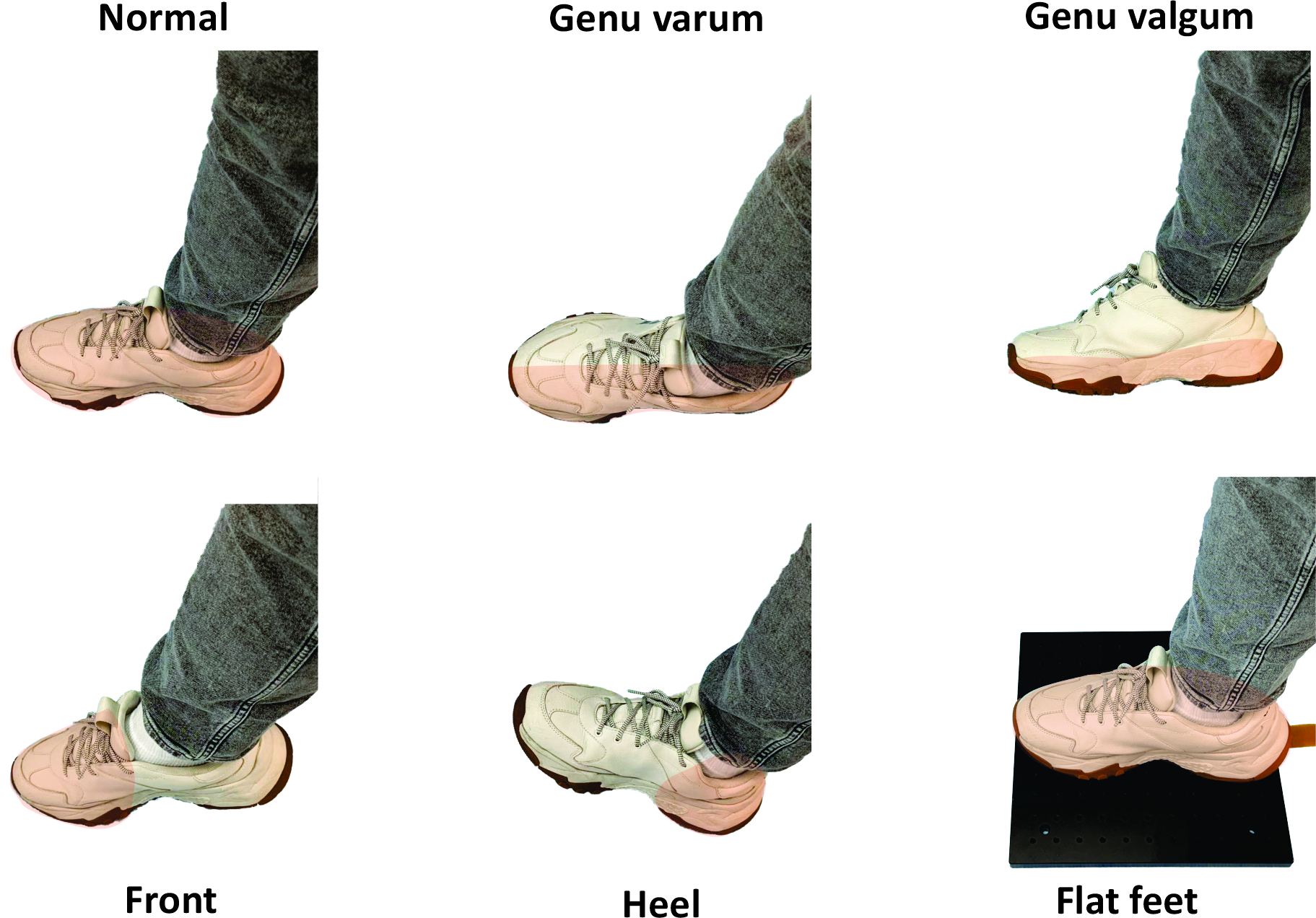


**Figure S24.** Different status of the volunteer for evaluation of the insole performance.


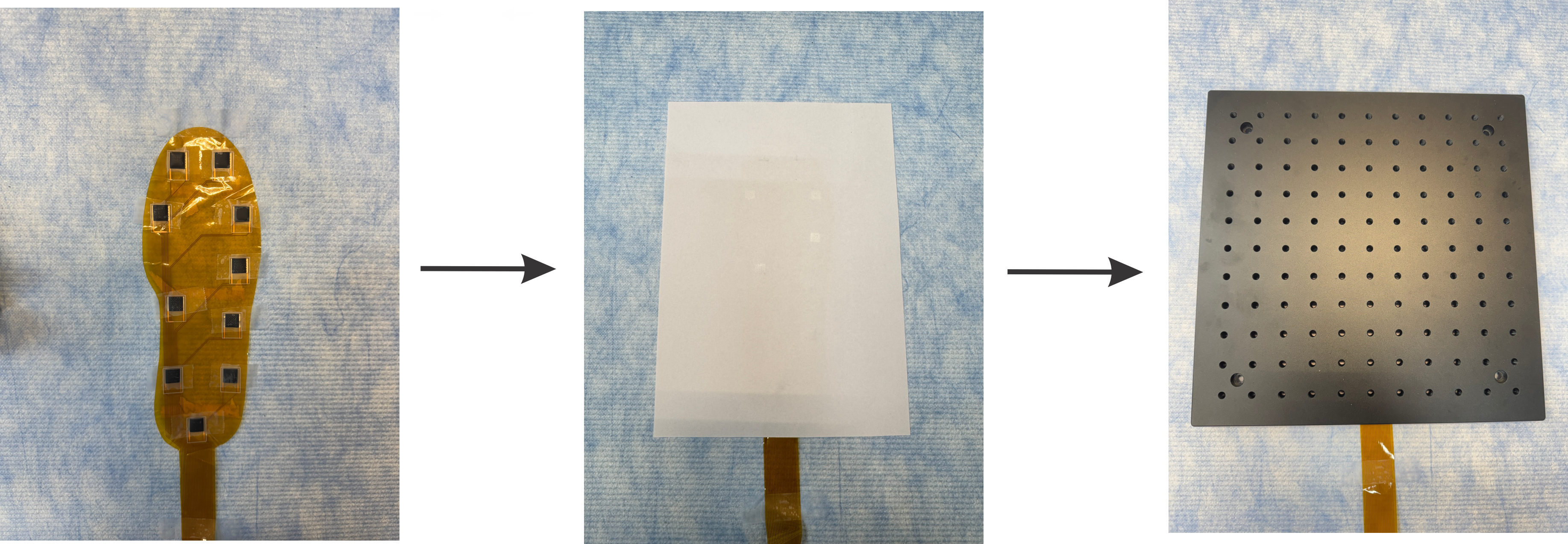


**Figure S25.** Testing scenario for simulation of flat feet.


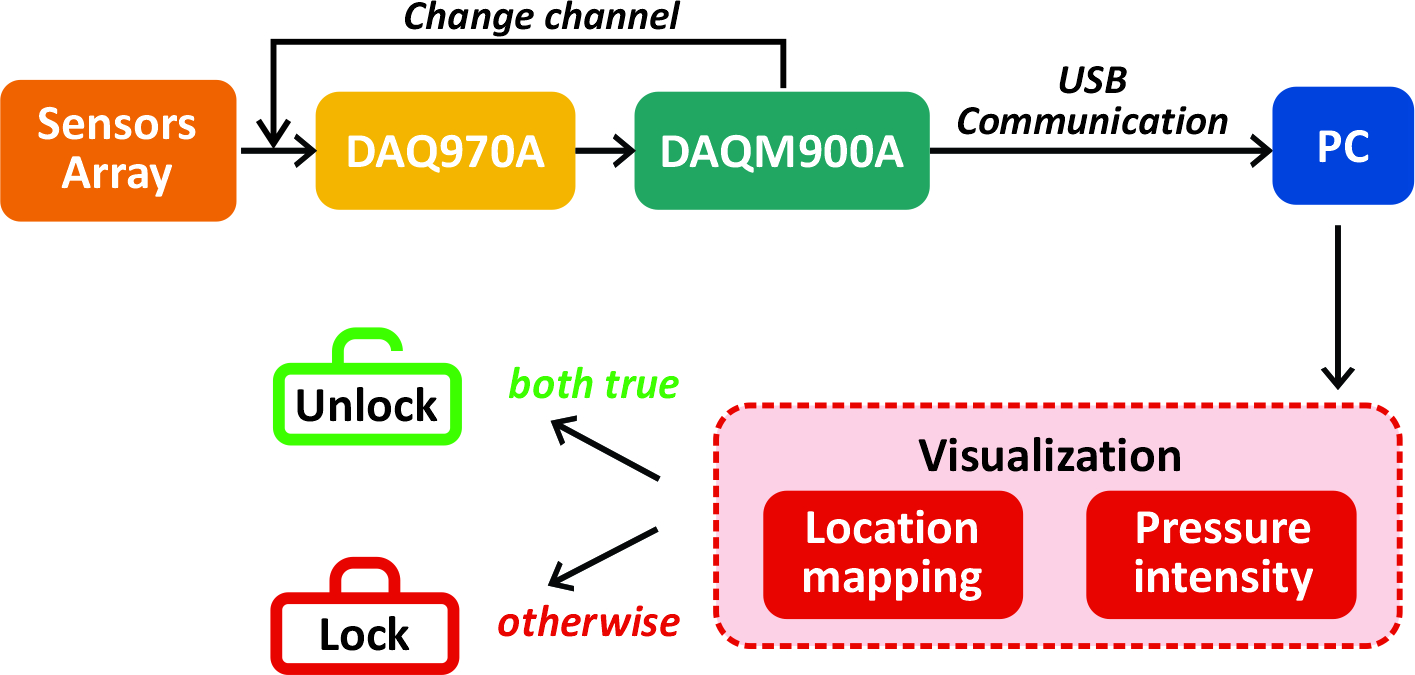


**Figure S26.** System architecture of the dual-factor authentication interface.

**Table S1.** Comparison results regarding the sensitivity and linearity range among our proposed sensor and other reported piezoresistive pressure sensors.

| Structure | Sensitivity and corresponding linear range | Detection limit [Pa] | | Electrode material | Conductive material | References |
| --- | --- | --- | --- | --- | --- | --- |
| Shark/Crocodile bio-inspired sensor architectures | 18.20 kPa⁻¹ (0–10 kPa) 1.10 kPa⁻¹ (10–40 kPa) 0.12 kPa⁻¹(40–80 kPa) | 1 Pa | | Cu/Silver paste | Graphene  /PDMS | [1] |
| Microvilli-Microstructures (MVMS) | 58.88 kPa⁻¹ (0.05–14.25 kPa) 2.93 kPa⁻¹ (14.25–200 kPa) 0.53 kPa⁻¹ (200–782.5 kPa) | | 50 Pa | Carbon cloth | PDMS-MWCNTs composite | [2] |
| Multi-layer structure | 3.997 kPa⁻¹ (4.7–100 kPa) | 4.7 Pa | | Silver ink | Ionic ink | [3] |
| Multi-level nano-microstructures (MLNM) | 3.94 kPa⁻¹ (0–117.5 kPa) | 10 Pa | | Copper tape | MXene nanosheets | [4] |
| Surface protruding structure | 2.32 kPa⁻¹ (0–100 kPa) | / | | / | PEDOT:PSS/CNC | [5] |
| Layered and microporous 3D structure | 0.298 kPa⁻¹ (25 Pa – 130 kPa) | 25 Pa | | Nickel-copper conductive fabric | Multi-walled carbon nanotubes | [6] |
| Hierarchical and interlocked microstructure | 15.01 kPa⁻¹ (0–35 Pa) 3.2 kPa⁻¹ (35–700 Pa) | 4 Pa | | Silver paste | Polypyrrole | [7] |
| Bionic intermittent structure | 461.27 kPa⁻¹ (0–42.8 kPa) 51.01 kPa⁻¹ (42.8–311 kPa) | / | | Silver paste | MXene (Ti₃C₂Tₓ) | [8] |
| Pyramidal carbon foam array + elastomer spacer | 24.6 kPa⁻¹ (0–1.4 MPa) | 1 Pa | | Ti/Au | Carbon foam | [9] |
| Sponge Structure | 0.0331 kPa⁻¹ (0.11–1250 kPa) | 110.83 Pa | | Copper foil | rGO | [10] |
| Pyramid microstructure | 0.71 kPa⁻¹ (1–80 kPa) 0.024 kPa⁻¹ (80–1000 kPa) | 1 Pa | | ITO/PET | PDMS  /MWCNTs | [11] |
| Carbon aerogel with lamellar-fibrous structure | 150 kPa⁻¹ (0–6.80 kPa) | 5 Pa | | Copper foil | GO/G Hybrid Conductive Filler | [12] |
| Porous structure | 96.1 kPa⁻¹ (up to 123.1 kPa) | 0.8 Pa | | Silver paste | PDMS/CNT | [13] |
| Microsphere spacer array | 2.3 kPa⁻¹ (100 Pa–7.5 kPa) 39.1 kPa⁻¹ (7.5–105 kPa) 11.7 kPa⁻¹ (105–160 kPa) | 100 Pa | | CNT | MXene (Ti₃C₂Tₓ)/ PDMS | [14] |
| Protrusion-curved surface sensing units with gradient heights | 8.24 kPa⁻¹ (98 Pa–70 kPa) 1.66 kPa⁻¹ (70–700 kPa) | 98 Pa | | / | MWCNT  /PDMS | [15] |
| Porous structure | 36.87 kPa⁻¹ (0–1.3 kPa) 11.39 kPa⁻¹ (1.3–60 kPa) 1.25 kPa⁻¹ (60–79 kPa) | 3.7 Pa | | Silver paste | MXene (Ti₃C₂Tₓ) | [16] |
| Porous structure | 15.4 kPa⁻¹ (up to 1.3 MPa) | 100 Pa | | Copper foil | Carbon black/ Carbon fiber | [17] |
| Dual-graded microstructure | 69.8 kPa⁻¹ (up to 300 kPa) | / | | Silver paste | MWCNTs | [18] |
| Multilayer step-like microstructure | 20.25 kPa⁻¹ (up to 35 kPa) | / | | Silver paste | PEDOT:PSS | [19] |
| Wrinkled microstructures | 1239 kPa⁻¹ (0–20 kPa) 74 kPa⁻¹ (20–100 kPa) | 5 Pa | | Copper tape | MWCNTs | [20] |
| Wrinkled microstructures | 1655 kPa⁻¹ (0.003–70.1 kPa) | 3 Pa | | Silver paste/ PET | SWNTs | [21] |
| Sandwich structure | 0.233 kPa⁻¹ (0–40 kPa) 0.048 kPa⁻¹ (40–120 kPa) | / | | PET | Magnetic graphene fibers | [22] |
| Porous structure | 1.62 kPa⁻¹ (0–29 kPa) 0.41 kPa⁻¹ (29–65 kPa) 0.10 kPa⁻¹ (65–200 kPa) | / | | Cu/Silver paste | rGO/ MWCNTs | [23] |
| Multilayer bionic structure | 3.54 kPa⁻¹ (0.015–100 kPa) 18.87 kPa⁻¹ (100–142 kPa) | 15 Pa | | Graphene/ Graphite conductive adhesive | Graphene/ Graphite | [24] |
| Porous structure | 0.7702 kPa⁻¹ (1–70 kPa) | / | | Copper foil | PDMS  /MWCNTs | [25] |
| Multilayered porous structure | 2602.26 kPa⁻¹ (0–0.47 kPa) 24.4 kPa⁻¹ (0.94–9.4 kPa) 6.95 kPa⁻¹ (18.8–141 kPa) | 2.16 Pa | | MXene sediment ink | MXene (Ti₃C₂Tₓ)/Zn²⁺ | [26] |
| Micropillar structure | 0.024 kPa⁻¹ (0–200 kPa) 0.04 kPa⁻¹ (200–450 kPa) | 5 Pa | | Silver paste | Ag | [27] |
| 3D interlocking structure | 21.67 kPa⁻¹ (3.4–43 kPa) | 3.4 Pa | | Ag/Cr | PANI/PS core–shell nanoparticles | [28] |
| Triple gradient microdomes | 974.1 kPa⁻¹ (0–1.8 MPa) | 1 Pa | | Cu/PI | CNT/PDMS | This work |

Supplementary References

[1]. B. Huang, J. Feng, J. He, W. Huang, J. Huang, S. Yang, W. Duan, Z. Zhou, Z. Zeng, X. Gui, ACS Appl. Mater. Interfaces 2024, 16, 19298.
[2]. H. Zhang, X. Chen, Y. Liu, C. Yang, W. Liu, M. Qi, D. Zhang, ACS Appl. Mater. Interfaces 2024, 16, 2554.
[3]. J. Liu, L. Wang, R. Xu, X. Zhang, J. Zhao, H. Liu, F. Chen, L. Qu, M. Tian, ACS Nano 2024, 18, 10818.
[4]. S. Wang, Y. Yao, W. Deng, X. Chu, T. Yang, G. Tian, Y. Ao, Y. Sun, B. Lan, X. Ren, et al., ACS Nano 2024, 18, 11183.
[5]. M. Zhu, C. Chen, A. Yu, Y. Feng, H. Cui, R. Zhou, Y. Zhuang, X. Hu, S. Liu, Q. Zhao, ACS Nano 2025, 19, 19488.
[6]. X. Wang, G. Wu, X. Zhang, F. Lv, Z. Yang, X. Nan, Z. Zhang, C. Xue, H. Cheng, L. Gao, Adv. Mater. 2025, 37, e2410312.
[7]. N. Bai, D. Xu, Z. Su, G. Li, L. He, Y. Chen, C. Guo, L. Zhou, X. Qin, J. Zhang, et al., Adv. Sci. 2025, e07135.
[8]. Y. Zhi, H. Zhang, L. Zhang, Q. Li, X. Kuang, W. Wu, Q. Zhou, P. Li, W. Li, H. Zhang, Adv. Fiber Mater. 2024, 7, 541.
[9]. J. He, R. Zhao, Z. Guo, Y. Li, Y. Zhao, L. Liang, S. Ji, H. Tian, Adv. Funct. Mater. 2025, 202510130.
[10]. J. Ma, B. Wen, Y. Zhang, R. Mao, Q. Wu, D. Diao, K. Xu, X. Zhang, Adv. Funct. Mater. 2025, 35, 202425774.
[11]. X. Meng, C. Zhang, H. Xie, S. Niu, Z. Han, L. Ren, Adv. Funct. Mater. 2024, 34, 202314479.
[12]. S. Wang, W. Deng, T. Yang, Y. Ao, H. Zhang, G. Tian, L. Deng, H. Huang, J. Huang, B. Lan, et al., Adv. Funct. Mater. 2023, 33, 202214503.
[13]. H. Xie, Z. Huang, J. Wan, R. Zhou, T. Chen, Y. Wang, D. Cheng, L. Yang, J. Ji, Y. Jiang, et al., Adv. Funct. Mater. 2024, 34, 202401532.
[14]. Z. Yu, C. Deng, J. Sun, X. Zhang, Y. Liu, C. Liu, F. Seidi, J. Han, Q. Yong, H. Xiao, Adv. Funct. Mater. 2024, 34, 202402707.
[15]. B. Jia, Z. Li, T. Zheng, J. Wang, Z.-J. Zhao, L. Zhao, B. Wang, J. Lu, K. Zhao, G. Luo, et al., Chem. Eng. J. 2024, 485, 149750.
[16]. A. Li, J. Xu, D. Xu, Z. Zhang, D. Cao, J. Li, W. Zhang, F. Zhang, Chem. Eng. J. 2025, 505, 159564.
[17]. Y. Tao, H. Zhang, J. Li, K. Shi, L. Jin, Y. Guo, J. Shi, Chem. Eng. J. 2025, 516, 163930.
[18]. Q. Xiang, H. Zhang, Z. Liu, Y. Zhao, H. Tan, Chem. Eng. J. 2024, 480, 147825.
[19]. Y. Yan, J. Zheng, Q. Zhang, Y. Li, G. Li, Z. Zhang, P. Wang, W. Wu, J. Zhai, Y. Xu, Chem. Eng. J. 2025, 517, 164221.
[20]. J. Zhang, J. Wu, L. Liu, X. Gu, X. Zhang, X. Li, Z. Shi, Y. Chen, J. Gao, J. Zhang, et al., Chem. Eng. J. 2025, 511, 162055.
[21]. J.-C. Shang, H. Yang, G.-Q. Hong, W.-H. Zhao, Y.-F. Yang, Composites, Part B 2023, 264, 110931.
[22]. H. Lu, B. Chen, X. Lai, H. Li, X. Zeng, Mater. Today Nano 2024, 28, 100512.
[23]. Z. Cui, Q. Hua, Y. Shi, R. Wei, Z. Dong, X. Dai, T. Huang, G. Shen, Z. L. Wang, W. Hu, Nano Energy 2025, 139, 110911.
[24]. T. Gong, H.-Q. Shao, X.-R. Sun, J.-X. Guo, J.-R. Hou, K. Ke, L. Gong, Y. Cao, Y.-H. Cao, R.-Y. Bao, et al., Nano Energy 2024, 120, 109173.
[25]. R. Chen, T. Luo, J. Wang, R. Wang, C. Zhang, Y. Xie, L. Qin, H. Yao, W. Zhou, Nat. Commun. 2023, 14, 6641.
[26]. P. Lu, T. Xu, Y. Yuan, X. Wang, W. Lian, C. Li, P. Yang, S. Guan, Polym. Compos. 2024, 46, 2710.
[27]. Z. Gao, Q. Zhang, Y. Wang, W. Zhang, J. Liu, D. Xu, X. Xia, S. Hu, B. Bian, Y. Zhao, et al., React. Funct. Polym. 2024, 196, 105840.
[28]. X. Yu, A. Adronov, Small 2025, 21, e2408795.
